# An integrative methodology to estimate high-resolution carbon stock and fluxes: a case study in the old-growth forests of the Chilean Patagonia

**DOI:** 10.1186/s13021-025-00381-6

**Published:** 2025-12-24

**Authors:** Taryn Fuentes-Castillo, Aarón Grau-Neira, Eduardo Morales-Santana, Deelan Rus-Valledor, David Trejo-Cancino, Adrián Pascual, Jorge F. Perez-Quezada

**Affiliations:** 1 Carbon Real, Apoquindo 7935, 903 Torre A, Las Condes, Santiago, Chile; 2 Department of Silviculture and Nature Conservation, Faculty of Forest Sciences and Nature Conservation, University of Chile, Av. Santa Rosa 11315, La Pintana, Casilla 9206, Región Metropolitana Santiago, Chile; 3 Département de Géographie, Université du Québec À Montréal, Montréal, H3C 3P8 Québec Canada; 4 Department of Geographical Sciences, University of Maryland, College Park, MD USA; 5 Department of Environmental Sciences and Renewable Natural Resources, Faculty of Agricultural Sciences, University of Chile, Santiago, Chile; 6 Institute of Ecology and Biodiversity, Barrio Universitario, Concepción, Chile; 7 Cape Horn International Center, Punta Arenas, Chile

**Keywords:** Carbon balance, Net ecosystem exchange (NEE), Evergreen forests, Forest carbon monitoring, Ecosystem flux modeling, Remote sensing integration

## Abstract

High-integrity carbon offset systems require scientifically robust and spatially explicit frameworks to quantify carbon pools and fluxes across ecosystems. We present an integrative methodology that combines eddy covariance measurements, airborne and satellite remote sensing, and modeling to extrapolate near real-time carbon flux monitoring to larger areas, using the old-growth temperate forests of Chilean Patagonia as a case study. Our approach delivers high-resolution aboveground biomass carbon density (30 m) and net ecosystem exchange (NEE, 30 m—30 min) estimates using flux tower data. By integrating ground-based flux measurements with high-resolution remote sensing, the proposed methodology constrains model parameters and spatial extrapolation, thereby reducing uncertainty relative to conventional inventory-based approaches. Our approach offers a replicable framework for informing climate policy, conservation planning, and emerging nature-based finance instruments while meeting operational needs in terms of scalability, technological integration, reproducibility, and traceability.

**Supplementary Information:**

The online version contains supplementary material available at 10.1186/s13021-025-00381-6.

## Introduction

The increasing concentration of greenhouse gases, particularly CO_2_, is a major driver of climate change, leading to significant environmental and socio-economic impacts globally [1]. Carbon offsets have been proposed as a mechanism to mitigate these effects by allowing entities to compensate for their impact through investment in projects that reduce and/or remove their emissions to the atmosphere [2]. Additionality is a critical aspect of these projects to ensure that the carbon (C) reductions would not have occurred without the execution of actions and projects [3]. REDD + (Reducing Emissions from Deforestation and Forest Degradation) is a prominent framework within this context, aimed at incentivizing forest conservation and sustainable management in developing countries to enhance C sequestration [4]. The integration of these strategies is essential towards achieving C neutrality and sustaining environmental policies to address climate change impacts.

While additionality remains a central criterion for C offset integrity, it has been criticized for not adequately reflecting the natural role of forests in removing atmospheric CO_2_ [5], as it may overlook their capability to keep large amounts of C sequestered [6]. This highlights the necessity of preserving existing irrecoverable C stocks in forests to prevent long-term emissions [7].

In addition, standard emission factors or the REDD + approach -based on traditional methods of assessing forest biomass stocking and fluxes- may fail to capture the full ecological contributions of natural forests. This is due to limitations in traditional sampling-based forest monitoring systems such as low-density national forest inventories (NFI) or to the omission of other C sinks present in the ecosystems but frequently ignored in calculations and in C budgets [8, 9]. It is therefore crucial to emphasize the role of well-preserved forests in mitigating climate change, which involves accurately measuring the amount of CO_2_ they remove from the atmosphere and the C stored across their biomass pools. This requires not only continuous forest monitoring technology but also a deeper focus on ecosystem health, forest conservation and management while ensuring flows of ecosystems services are recognized [10], as part of an integrative approach needed to understand and enhance the C sequestration potential of natural forests [11].

To address these challenges, innovative technologies have emerged. Among them, LiDAR (Light Detection and Ranging) technology has revolutionized the mapping of forests and C baselines, enabling a precise three-dimensional sensing of vegetation structure and estimated biomass [12]. Temporal series of LiDAR measurements inform on change where growing and disturbance dynamics in forests occur fast, but for old-growth forests or peatlands changes in structure and C stocks are not that tangible from laser pulses. For this reason, eddy covariance systems emerge as a vital tool for measuring near real-time CO_2_ fluxes, providing critical insights into C exchange between ecosystems and the atmosphere, and complementing structural biomass assessments. This method involves the use of high-frequency measurements of gas concentration and 3D wind direction, allowing for accurate estimations of gas exchange between the biosphere and the atmosphere [13, 14]. The integration of these technologies not only enhances our understanding of C sequestration processes but also informs conservation strategies by identifying key areas for protection and restoration [15]. These technologies represent powerful tools for advancing our knowledge of C management and ecosystem conservation [16].

With the aim of generating an integrative methodology to estimate high-resolution C stock and flux estimations in vast areas of natural ecosystems, we worked in the old-growth forests of the Chilean Patagonia with the following objectives: (1) generate a spatial delimitation of vegetation areas that are functionally similar, (2) estimate the C content in aerial biomass with high spatial resolution, (3) model the historical net ecosystem exchange of CO_2_ (NEE), and (4) extrapolate high temporal resolution on-site measurements of NEE to larger areas. Through these efforts, our proposed methodology is useful to evaluate the contribution of natural and managed ecosystems to climate change mitigation, while strengthening biodiversity conservation.

## Methods

### Spatial datasets utilized in this study and methods overview

The spatial datasets used in this study are summarized in Table 1, which were selected to comprehensively capture key aspects of forest C sequestration. To integrate these data sources, we applied several preprocessing and harmonization procedures. Detailed descriptions of their application are provided in the following sections, which address the establishment of a baseline of C stocks and the near real-time measurement of CO₂ fluxes using eddy covariance techniques, in conservation easement lands.

**Table 1:** Overview of spatial datasets utilized in this study and the stage of the methodology where they were used, as described in Fig. 1

Variable	Source	Temporal range	Native scale	Native resolution	Processed resolution	Reference	Stage
Digital elevation model	DEM Alos Palsar	2006—2011	1:25,000	12.5 m	30 m	[17, 18]	a
Vegetational functional type	Vegetational belts	2018	1:50,000	-	30 m	[19]	a
Canopy height model	High Resolution Canopy Height Maps by WRI and Meta	2017—2020	-	1 m	30 m	[20]	b
Surface reflectance and brightness temperature data HLSL30	HLS (Harmonized Landsat Sentinel-2)	2013—2020	-	30 m	30 m	[21]	c1
Solar induced fluorescence	Reconstructed TROPOMI Solar Induced Fluorescence	2001—2019	-	5 km	30 m	[22]	c1
Gross primary production	Global GPP (Gross Primary Production) data	2001—2019	-	5 km	30 m	[23]	c1

**Fig. 1:**
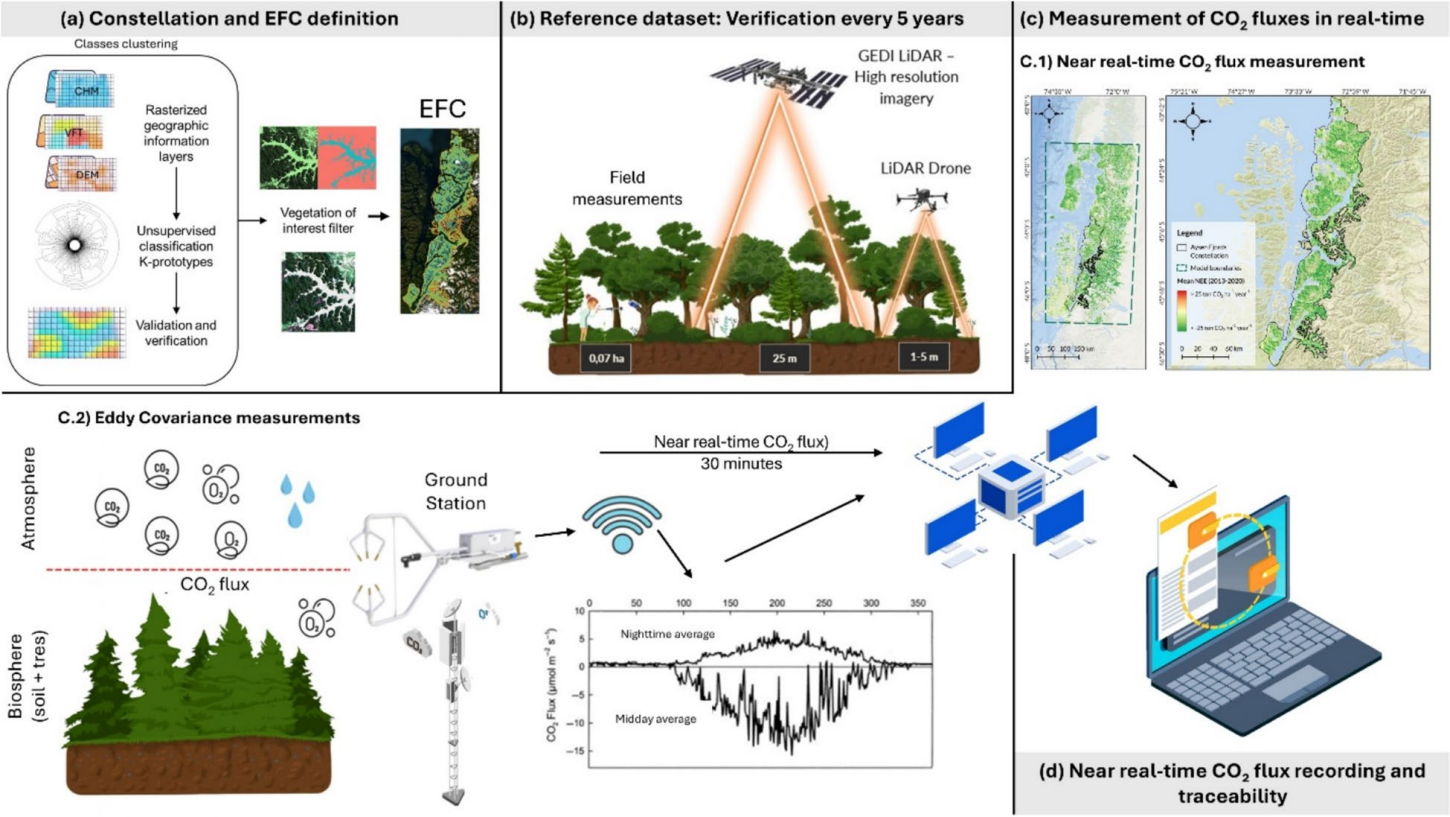
Research workflow outlining the study’s sequential processes: (**a**) Constellation and Eco Functional Cluster (EFC) definition; (**b**) Establishment of a structural reference dataset with re-verification every 5 years; (**c**) Near real-time CO₂ flux measurement—including (**c.1**) spatial extrapolation of Net Ecosystem Exchange and (**c.2**) Near real-time Eddy Covariance analysis; and (**d**) Near real-time CO₂ flux recording and traceability

To provide an overview, our methodology follows five sequential stages, summarized in Fig. 1: (a) compilation and preprocessing of multi-source spatial datasets; (b) delineation of eco-functional clusters (EFCs) representing areas with similar biosphere–atmosphere exchange dynamics; (c) estimation of aboveground biomass carbon density (AGCD) using LiDAR-derived canopy structure; (d) measurement and spatial extrapolation of near real-time CO₂ fluxes through the eddy covariance technique; and (e) integration of these layers into an operational carbon accounting framework. This workflow enables a consistent linkage between ground-based measurements, remote sensing products, and property-level carbon accounting.

Each dataset listed in Table 1 was processed to ensure compatibility across spatial and temporal scales. Original data were harmonized through coordinate standardization (UTM Zone 18S, EPSG: 32718) and aggregated into common temporal intervals (8-day or annual) according to variable availability. Datasets associated with stages A and B—used respectively for the definition of Eco-Functional Clusters (EFCs) and for the generation of the reference dataset based on LiDAR and field measurements—were resampled to a uniform 30-m spatial resolution to maintain spatial consistency across inputs. In contrast, datasets used in stage C, corresponding to the extrapolation modeling, originally had coarser resolutions (e.g., 5 km) and were therefore downscaled to 30 m using auxiliary predictors derived from satellite-based vegetation indices and surface variables. These preprocessed datasets were then integrated according to the corresponding methodological stage described in Fig. 1, while stage D refers to data management and traceability processes that ensure continuous CO₂ flux monitoring and system reliability. Each of these stages is detailed in the following sections, providing a step-by-step description of the integrative workflow.

### Study area: the Chilean Patagonia

The Chilean Patagonia, recognized as the largest system of estuaries and fjords in the Southern Hemisphere, encompasses a total area of 452,204 km^2^, including both inland sea and terrestrial landscapes [24]. Specific study area for this work was named the Aysen Fjords Constellation (43° 31′ 59.545" S and 46° 38′ 1.386" S; Fig. 1, left panel) and is located in the Aysén region in Chile and encompasses the Aysen fjords, the Yacaf channels, Magdalena Island, Quitralco Fjord, Exploradores Bay, and extends to San Rafael Lagoon.

### Definition of eco-functional clusters

**Fig. 2:**
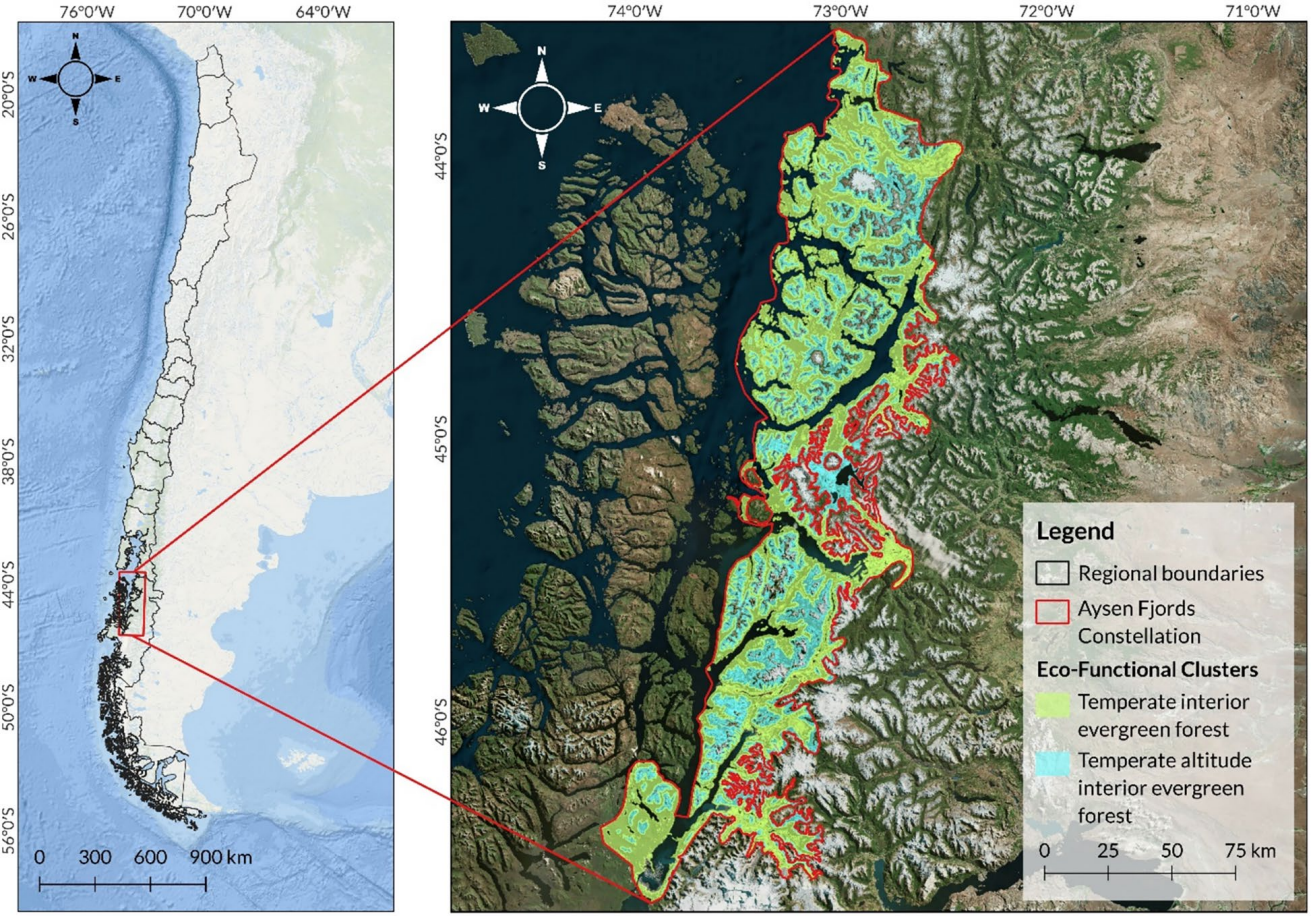
Study area. Left panel shows the Chilean Patagonia; Right panel shows the Aysen Fjords Constellation

To extrapolate CO_2_ flux measurements requires the identification of regions with statistically similar biosphere–atmosphere C exchange dynamics, which we call Eco-Functional Clusters (EFCs). The EFCs were defined using the unsupervised k-prototypes classification algorithm [25], which incorporates both categorical and continuous variables (Table 1): (1) the functional characteristics of vegetation, based on the description of vegetation types proposed by Luebert and Pliscoff [19], which were grouped according to the predominant vegetation formation, macroclimate, and type of continentality,(2) terrain elevation (derived from ALOS–PALSAR, 2021); and (3) a wall-to-wall canopy height product developed by Tolan et al. [20], which integrates GEDI sampling, airborne LiDAR, and high-resolution optical data into a 1-m resolution surface. These three datasets were individually aggregated to a 30-m grid and stacked to create a multivariate data set comprising 130 million observations across the Chilean Patagonia. We used 30 repetitions of 10,000 draws (data points) each to determine the optimal number of clusters based on Silhouette scores [26]. A training dataset of 1 million valid data points was used for creating the EFC classification, after which the distribution of the input variables was assessed to secure coherence – statistically and spatially- in the resulting classes. To define operational units for implementation and monitoring, EFCs were spatially grouped into administrative units called ‘constellations’, defined as continuous geographic areas > 50,000 ha containing from 1 to 6 EFCs. The 10-m global land cover product from the European Space Agency [27] was used to isolate forest classes from non-forest lands (agriculture, water bodies, etc.) and to correct the proportion of forest areas within a given EFCs. The analyses were performed in R using R Studio [28] and the *clustMixType* library [25], while visualization was done with R and QGIS 3.34.1. All spatial analyses used the UTM Zone 18S projection (EPSG:32718). Vegetation types were grouped according to their functional ecological characteristics, which include similarities in vegetation formation, macroclimate, and biogeographic context (Table S1). These functional classifications were integrated as core variables in the delineation of EFCs—spatial units that represent regions with statistically similar biosphere–atmosphere C exchange dynamics. A schematic overview of the EFCs procedure is presented in Fig. 2a.

### Reference conditions to define areas for C stock and fluxes in conservation easement’s lands

In this study, we evaluated lands in Chilean Patagonia whose owners agreed to include their properties in the Constellation study area under a conservation commitment. Landowners wishing to enroll their properties in the program must sign a Conservation Easement (DRC for its name in Spanish: ‘*Derecho Real de Conservación*’), which commits them to refrain from any intervention for at least 25 years that could harm biodiversity or reduce the initial forest C level and annual C sequestration. Because the DRC establishes an absolute conservation function, infrastructure development is strictly prohibited. However, landowners may choose to place only a portion of their property under the DRC agreement, allowing them to exclude specific areas where construction or other activities may be permitted.

Carbon accounting projects require a baseline of the aboveground biomass or C to assess the conditions of the forest before flux measurements start. Our preliminary estimation of forest C storage is based on LiDAR-derived forest structural metrics (Fig. 2b). In favorable weather conditions, our methodology employs Unmanned Aerial Vehicles (UAV) coupled with LiDAR sensors and/or LiDAR-mounted systems in wider-range aircrafts – commonly referred to as Airborne Laser Scanner (ALS) - using 3D point clouds data collected at a minimum of 10 points (laser pulses) per square m. for a precise retrieval of altimetric information and complex topography. When adverse weather conditions constrain or restrict the use of UAV-LiDAR or ALS-LiDAR, the canopy height model (CHM) from the World Resources Institute (WRI) and META [20] defined for the 2017–2020 period is integrated. Our LiDAR acquisitions and the existing global CHM map provide a baseline of forest structure and forest C biomass for comparisons over time and the presence and magnitude of degradation and the disturbances causing degraded conditions in forests.

After incorporating input data on forest structure and canopy height, we applied the allometric equation proposed by Pascual et al. [29] to derive aboveground biomass density (AGBD, Mg ha⁻^1^) using estimates of CHM (see Sect. "Definition of eco-functional clusters"), which represent average top-of-canopy conditions:

1 $$AGBD = 1.4535\times CH{M}^{1.5546}$$

where *AGBD* is the aboveground biomass density and *CHM* is the canopy height model (m). We then applied a conversion factor of 47%, as established by the IPCC (2006) to estimate the C fraction in the forest biomass:

2 $$AGCD = AGBD\times 0.47$$

where *AGCD* is the aboveground C density (Mg C ha⁻^1^).

### Measurement and spatial extrapolation of CO_2_ fluxes

The estimation of net CO₂ fluxes in this model relies on continuous measurements between the ecosystem and the atmosphere using the eddy covariance (EC) technique (Fig. 2c). This methodology is well-supported by scientific literature and recognized by international climate research networks [14]. Numerous studies have demonstrated the efficacy and precision of EC in quantifying gas exchanges between the atmosphere and forest ecosystems [30–35]. The global acceptance of this technique stems from its ability to provide continuous measurements at fine temporal scales, thereby enhancing the understanding of gaseous exchange processes and facilitating the monitoring of CO_2_ fluxes in forest environments. The EC technique quantifies gas and heat exchange fluxes between the Earth’s surface and the atmosphere, focusing on carbon dioxide (CO_2_), water vapor (H_2_O), and sensible heat. High-frequency sensors are employed to continuously measure fluctuations in CO_2_ concentration, water vapor and wind direction and speed, along with meteorological variables such as solar radiation, air temperature and humidity. The technique operates on the analysis of rapid, random fluctuations (or eddies) in the atmosphere caused by air movement across various scales. Flux calculations are based on the covariance of fluctuations between CO_2_ concentration and vertical wind speed relative to their means [36]. In this case, flux data were collected for the period 15 July 2024–15 July 2025 at 10 Hz (10 measurements per second) using a closed-path infrared gas analyzer (LI-7200RS, LI-COR, Lincoln, Nebraska, USA) and a 3-D ultrasonic anemometer (Windmaster, Gill Instruments, Lymington, UK). The high-frequency data were subsequently averaging over 30-min intervals.

High-frequency raw data from the eddy covariance system were processed in EddyPro (LI-COR Biosciences, version 7.0.9) using advanced corrections mode. Processing included coordinate rotation to correct for sensor tilt, detrending to compute turbulent fluctuations and time-lag compensation between wind and gas measurements. Density effects were evaluated with the Webb-Pearman-Leuning correction, but no adjustment was required for CO_2_ fluxes because the closed-path LI-7200RS analyzer inherently compensates for such fluctuations. Despiking and statistical screening were performed following Vicker & Mahrt (1997), and spectral corrections were applied to account for both high-pass filtering effects [37] and low-pass attenuation [38].

Post-processing involved the removal of physically impossible values and fluxes with a quality flag of 2 in the Mauder and Foken [39] classification, addition of storage terms to CO_2_ fluxes, and filtering of low-turbulence period using the u* threshold method used in Papale et al. [40]. Fluxes with a strong influence of areas outside the target footprint were excluded following the Kljun et al. [41] footprint model. Time series gaps were filled using a bagging regression model [42, 43]. This approach, similar to the methodology proposed by Zhu et al. [44] for addressing longer gaps, utilizes a random forest as the base estimator. This method effectively fills in missing data while accounting for the complex, nonlinear relationships within the time series. All processing steps were applied uniformly to ensure standardization and reproducibility.

According to the convention, negative fluxes indicate CO_2_ uptake by the ecosystem, while positive fluxes signify CO_2_ emissions from the ecosystem to the atmosphere. The list of instruments and corrections applied to the EC data, along with detailed calibration, configuration, and data post-processing, are documented in the Supplementary Table S2 and Text S3.

As the footprint of the EC tower is limited to a few hundred meters, CO₂ flux measurements were spatially extrapolated to the broader landscape using remote sensing and modeling approaches. These methods are described in detail in Supplementary Material S4. Briefly, gross primary productivity (GPP) was modeled based on satellite-derived solar-induced chlorophyll fluorescence (SIF), while ecosystem respiration (RECO) was estimated using land surface temperature (LST) and the land surface water index (LSWI). The NEE was calculated as the difference between RECO and GPP. The predictiveness of the satellite-based products was validated using EC flux data at the Senda Darwin Forest site (https://ameriflux.lbl.gov/sites/siteinfo/CL-SDF), allowing the generation of GPP, RECO, and NEE maps at 30-m spatial resolution based on 8-day satellite observation intervals for the period 2013–2020. The NEE at the reference site was partitioned into RECO y GPP following the standard method proposed by Reichstein et al. [45].

To integrate these data into C accounting at the scale of individual land properties, a spatial extrapolation factor (PExt) was calculated. This weighting factor compares the median NEE values within the EC tower footprint and those across the entire eco-functional cluster (EFC). It enables the adjustment of modeled flux values for properties not directly measured by the EC system and is updated every six months to reflect the changing extent of registered properties (Eq. 3):

3 $${P}_{Ext}=\frac{Q2\left(NE{E}_{EFC}\right)}{Q2\left(NE{E}_{f}\right)}$$

where *P*_*Ext*_ is the spatial extrapolation weighting factor, *Q2*(*NEE*_*EFC*_) is the 2nd quartile or median of NEE (2013–2020) data for land properties within the EFC, and Q*2*(*NEE*_*f*_) is the 2nd quartile or median of NEE (2013–2020) data for the EC tower footprint.

This multi-source approach integrates airborne LiDAR for structural baseline mapping, EC towers for reference flux data, and satellite imagery to extrapolate fluxes over the landscape. LiDAR-derived canopy height and cover inform the spatial characterization of forested areas; EC flux data serve as calibration references for GPP and RECO models; and satellite products (e.g., SIF, LST, LSWI) allow spatiotemporal generalization. Together, these layers are harmonized through the Eco-Functional Cluster framework to produce traceable, high-resolution maps of C stocks and fluxes across properties.

### Framework for near real-time CO₂ flux recording and traceability

We developed a framework for recording near real-time CO₂ fluxes at the level of individual land properties, with the objective of generating continuous datasets that could support C accounting where relevant. This process establishes the entry date of each land property into the system and estimates the net CO₂ sequestration achieved over time. The spatial extent of each EFC within registered properties is verified using LiDAR-based forest canopy metrics or high-resolution satellite imagery (< 5 m pixel) to ensure the accuracy of land cover classifications and baseline conditions.

Although each component (LiDAR, eddy covariance, and satellite-based modeling) was processed independently using its own tailored protocols, the outputs were integrated at the land property level to evaluate net CO₂ fluxes and carbon sequestration potential. This operational integration allows for spatially explicit carbon accounting that reflects both direct ecosystem measurements and their scalable extrapolation. While no formal cross-calibration was performed between methods, their combined application improves internal consistency, supports verification across independent sources, and enhances the practical applicability of the framework in C management contexts.

The use of a C flux registry is recommended to track cumulative CO₂ fluxes at both the Constellation-study area and individual property levels (Fig. 2d). The registry provides a structured record of NEE, where the absorption of one ton of CO₂ by the ecosystem is logged as a unit entry. Each record includes detailed information such as gross CO₂ fluxes (total absorbed or emitted), net contributions and the cumulative flux balance at the site.

The registry also documents the size of the productive area (land actively contributing to net CO₂ removal), associated land cover types, and the monitoring stations linked to each entry. Additionally, any residual or unallocated CO₂ fluxes are retained for future validation or recalibration purposes.

If C accounting is developed to generate C credits, the entire registry should be secured through a blockchain-based platform, which ensures data immutability, transparency, and traceability for scientific use and independent verification, while maintaining a transparent record of data revisions to support reproducibility and independent validation.

To evaluate the accuracy of our estimates or C stock and fluxes, we calculated indicators of accuracy (i.e., coefficient of determination and root mean square error) for tree height, forest biomass, and the spatial extrapolation of on-site C fluxes.

Uncertainties in the C flux estimates were quantified by combining the contributions of three main sources: random error, gap-filling and friction velocity filtering. Random errors were estimated following Lasslop et al. [46], which derives the standard deviation (SD) of flux differences under similar meteorological conditions to represent the stochastic variability inherent in these measurements. The uncertainties associated with u* filtering and gap-filling were assessed using a bootstrap, repeated 100 times. For each iteration, the resulting 30-min NEE series was recalculated,then, the SD across the 100 iterations was used to quantify these sources of uncertainty. The SD of weekly and annual fluxes was estimated by propagating the uncertainties assuming independence from each other, thus we were able to add the variances of each one and calculate the square root of the total. The result was then expressed in Mg CO₂ ha⁻^1^ year⁻^1^.

## Results

### Aysen fjords constellation and baseline

Within the Aysen Fjords Constellation, two EFCs were defined: the Temperate Interior Evergreen Forest (412,838 hectares) and the Temperate Altitude Interior Evergreen Forest (280,897 hectares) (Fig. 1).

**Fig. 3:**
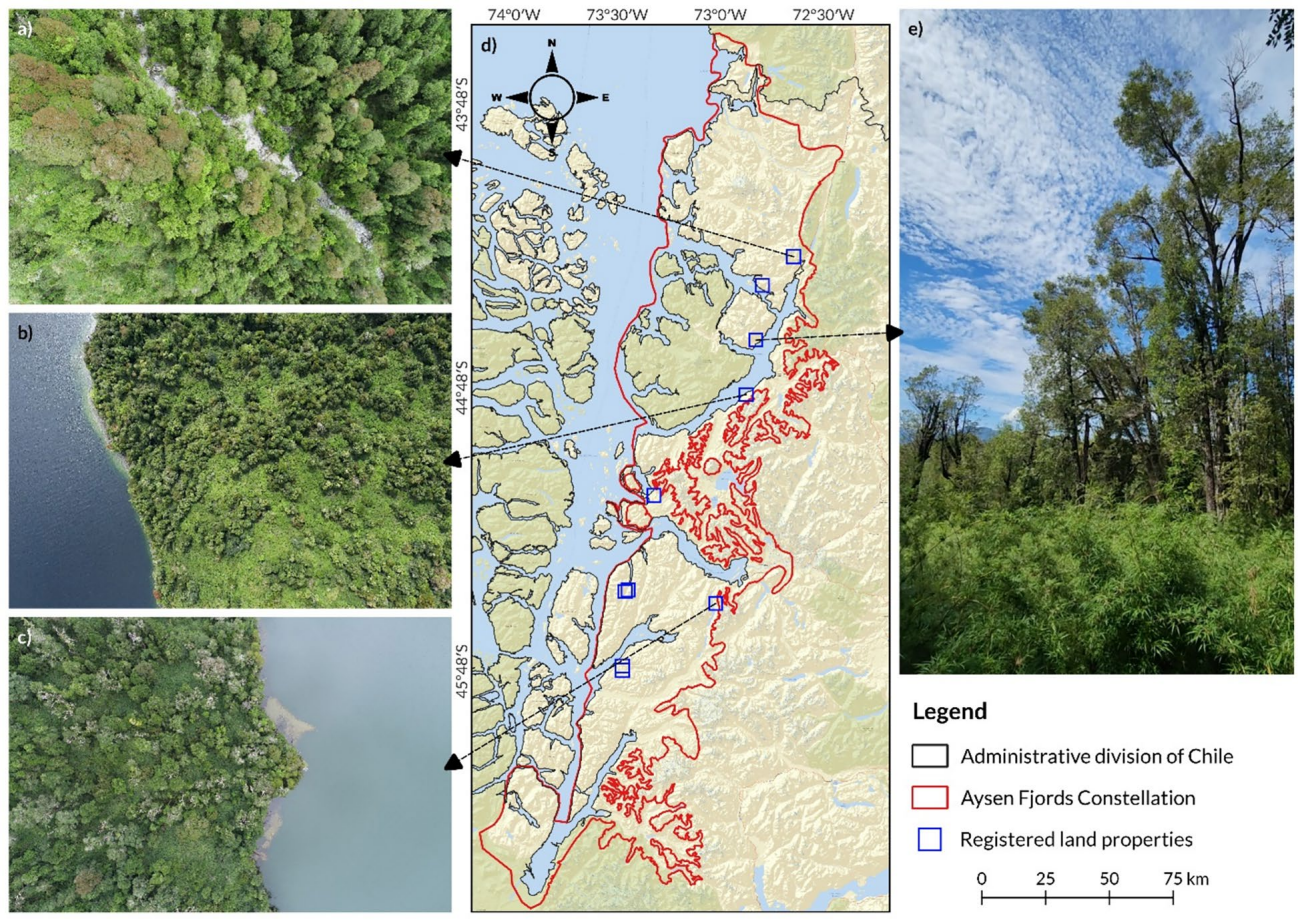
Conservation Easement’s land properties considered in this study. Panels (**a**), (**b**), and (**c**) display aerial views captured by a drone, showcasing the Puyuhuapi Evergreen Forest. Panel (**d**) illustrates the boundaries of the constellation and provides a reference location for the registered properties. Panel (**e**) presents the vegetation of the Isla Magdalena area

As of November 2024, the platform includes nine properties that are registered in the system, covering a total area of 8,913 hectares. Out of this area, 4,390 hectares are covered by EFC_1, while 2,131 hectares are covered by EFC_2. The primary vegetation types identified in these areas correspond to the Puyuhuapi Evergreen Forest and the High Montane Deciduous Shrubland (Gajardo, 1994). The location of the active properties and the predominant vegetation type can be seen in Fig. 3.

Regarding the LiDAR evaluation, general statistical results encompass both the UAV-LiDAR data at a 5-m spatial resolution (Supplementary Material S5) and the META-WRI data resampled to the same resolution, pertaining to the land properties analyzed with satellite images. CHM indicates that the conservation areas have an average tree height of 11.7 m (median: 11.36 m), with the tallest trees reaching up to 59.9 m. AGBD estimates averaged 90.1 Mg ha⁻^1^ (median: 74.3 Mg ha⁻^1^) across the nine land properties.

Table 2 presents the descriptive statistics for canopy height and aboveground biomass density (AGBD), including values from the full dataset and disaggregated by source (UAV and META-WRI). All AGBD values were derived from pixels with tree height ≥ 4 m, in accordance with the biomass estimation threshold. The frequency distributions of these variables are shown in Supplementary Figure S7. The relationship used to convert canopy height into aboveground biomass was derived from Chilean National Forest Inventory (NFI) data and is illustrated in Supplementary Figure S6. This figure shows the fitted regression model (AGBD = 1.453 × Height^1.555^), which had an R^2^ of 0.43 and a root standard error (RSE) of 106.7 Mg ha⁻^1^.

**Table 2:** Descriptive statistics of canopy height model (CHM, in meters) and aboveground biomass density (AGBD, in Mg ha⁻^1^) for the study area

Statistics	CHM			AGBD		
	General	UAV	META-WRI	General	UAV	META-WRI
Min	0	0	0	12.54	12.54	12.54
1 st quantile	7.00	10.63	6.16	44.80	76.84	40.44
Median	11.36	17.32	10.21	74.31	135.27	63.64
Mean	11.70	16.77	9.82	90.06	144.24	68.16
3rd quantile	15.65	23.00	13.67	113.33	198.27	90.10
Max	59.99	59.99	34.70	844.44	844.44	360.61

Values are presented for the general distribution and for two sources of canopy height data: drone-based UAV CHM and the META-WRI product

### Carbon flux measurements and spatial extrapolation

During the one-year period of CO_2_ flux measurements, 92% of the 30-min data periods were registered. After applying all the quality filters, 73.5% of the periods were classified as good quality data. The other 26.5% of the periods were filled using a machine-learning model (bagging regressor), which had an R^2^ = 0.59.

**Fig. 4:**
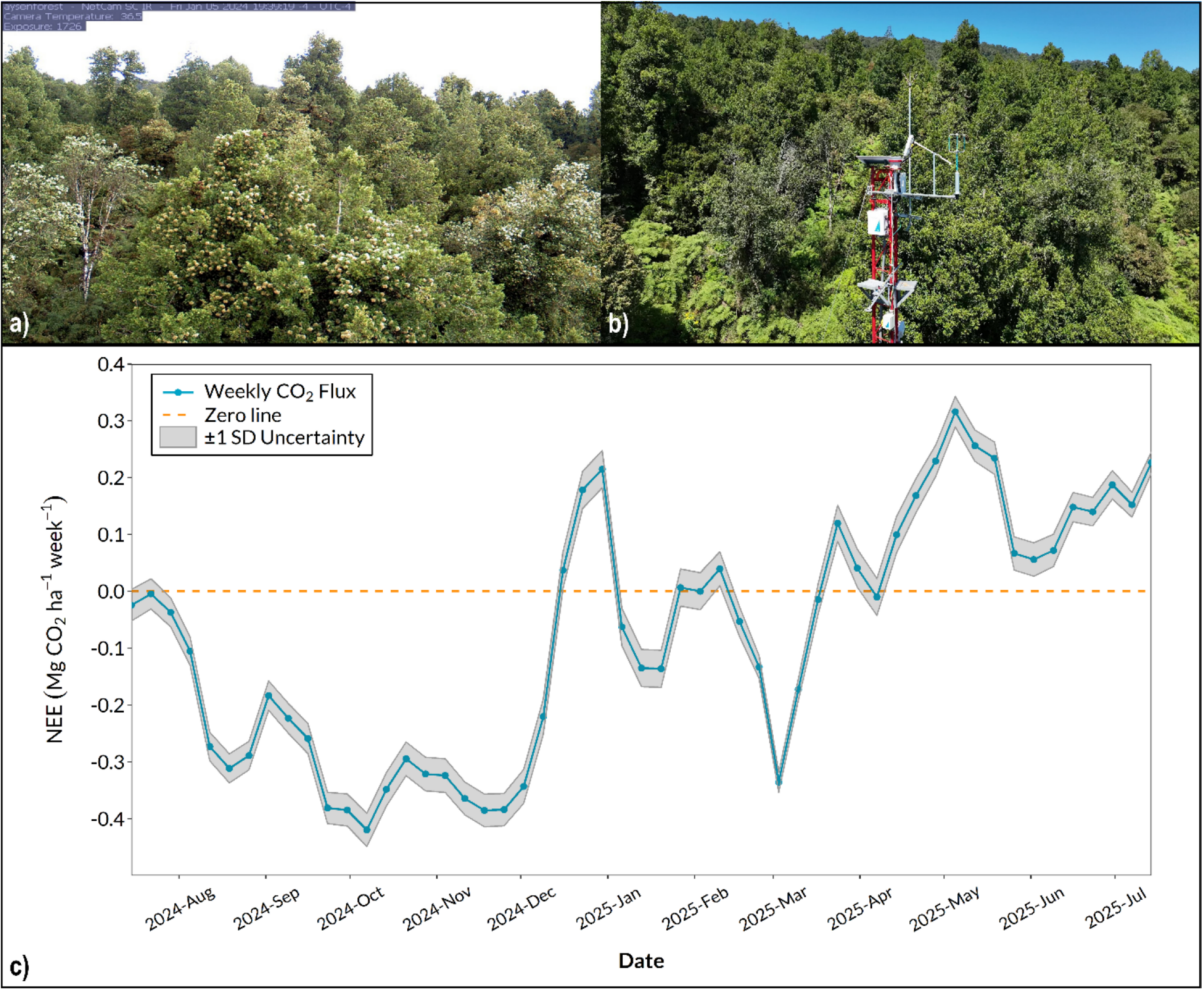
Eddy covariance system deployed at the Aysén Fjords Constellation for real-time CO₂ flux monitoring: (**a**) PhenoCam image of the old-growth evergreen forest at the study site; (**b**) Eddy covariance tower used for continuous measurements of net CO₂ exchange between the ecosystem and the atmosphere; (**c**) Weekly cumulative CO₂ fluxes (Mg CO₂ ha⁻^1^ week⁻^1^) from 15 July 2024 to 15 July 2025. The solid blue line shows post-processed fluxes, while the shaded grey band indicates the propagated uncertainty (± 1 standard deviation, SD); both values represent 3-week moving averages

Figure 4 (panels a and b) shows the forest and eddy covariance tower deployed at the Aysen Fjords constellation, while Fig. 4c displays the weekly cumulative CO_2_ fluxes for the study period and the associated SD. The annual NEE of the forest during this period was −4.0 Mg CO2 ha^−1^ year^−1^. The propagated annual uncertainty, incorporating u* filtering, gap-filling, and random error resulted in a SD of 0.206 Mg CO_2_ ha^−1^ year^−1^.

Supplementary material S8 shows the footprint area, i.e., the spatial delimitation of areas that effectively contribute to gas flux data generation (CO_2_ and H_2_O), where it can be observed that 70% of the measured flux is contained within 17.4 hectares.

The SIF-GPP model showed a strong fit across all seasons, with coefficients of determination (R^2^) ≥ 0.8. The highest accuracy was observed in summer (GPP = 18.2 * SIF, R^2^ = 0.93, RSE = 1.88 g C m⁻^2^ day⁻^1^), followed by spring (GPP = 15.9 * SIF, R^2^ = 0.90, RSE = 1.79), autumn (GPP = 13.5 * SIF, R^2^ = 0.81, RSE = 0.99), and winter (GPP = 10.4 * SIF, R^2^ = 0.80, RSE = 0.68). For the RECO model, the nonlinear least-squares regression yielded an R^2^ of 0.54, with an RSE of 2.80 g C m⁻^2^ day⁻^1^. The fitted parameters included R₀ = 2.23, k = 0.68, LSWImax = 0.87, and E₀ = 198.31.

**Fig. 5:**
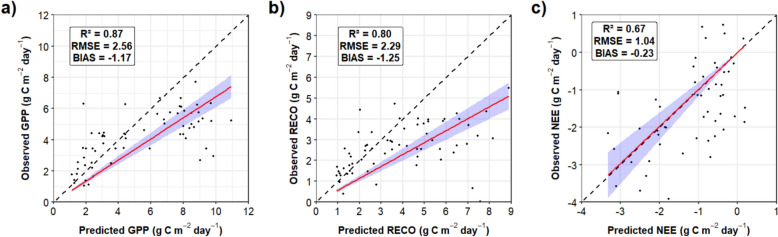
Predicted vs. observed values in a temperate evergreen forest ecosystem in the CL-SDF Senda Darwin station for (**a**) GPP, (**b**) RECO and (**c**) NEE satellite modeling (2013–2020). The black dashed line represents the identity line (1:1), while the red line represents the linear fit constrained to pass through the origin (intercept-constrained regression), assuming that predicted values are zero when observed values are zero. Shaded areas indicate the 95% confidence interval of the fit

Figure 5 presents the validation of satellite-based flux models against EC tower observations at the CL-SDF site, with panels showing predicted versus observed values for GPP (a), RECO (b), and NEE (c). The models exhibit strong predictive performance for GPP and RECO (R^2^ = 0.87 and 0.80, respectively), and moderate agreement for NEE (R^2^ = 0.67), with residuals distributed symmetrically and low bias across flux components. RMSE and bias values are reported within each panel. All models were fitted using an intercept-constrained linear regression and filtered for outliers via Hampel filtering.

**Fig. 6:**
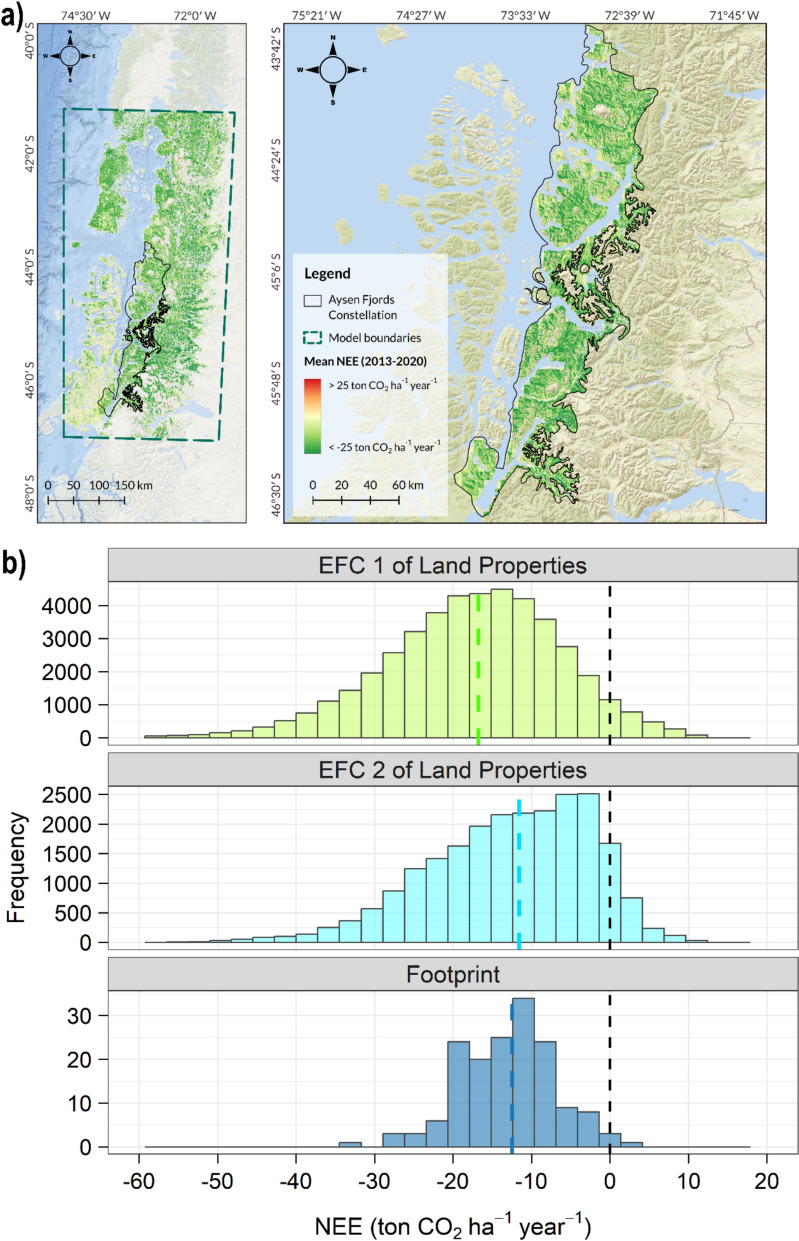
(**a**) Historical mean NEE within the Aysen Fjords Constellation from 2013 to 2020, (**b**) histograms of historical mean NEE data (2013–2020) for the eddy covariance system 1 footprint and the land properties located at eco-functional clusters (EFC) 1 and 2. The segmented lines represent the median of each data population according to their respective color, while the black one represents net zero. For NEE, negative values indicate that the forest behaves as a CO_2_ sink, while positive values signify CO_2_ emissions

Figure 6-a illustrates the historical mean NEE within the Aysen Fjords Constellation, determined by the difference between the historical means of GPP and RECO for the period from 2013 to 2020, showing a variability range of –15.2 ± 11.5 ton CO_2_ ha^−1^ year^−1^ (mean ± SD). Figure 6-b presents the distribution histograms of NEE data from the footprint of the eddy covariance system, compared to NEE data from land properties located at EFC_1 and EFC_2. The segmented lines on the x-axis indicate the median of the data, while the bars are stacked to enhance visualization. These graphs provide insights into the distribution range of the eddy covariance system measurements in relation to the distribution spectrum of data from the land properties within the Aysen Fjords Constellation.

Based on the footprint results (representative polygon) of the CO_2_ flux measurements, the weighting factors for spatial extrapolation for EFC_1 and EFC_2 were 1,233 and 0,851, respectively. The total count of C accounting from July 15, 2024, 00:00:00 to November 1, 2024, 00:00:00 is 21,218 units of net ecosystem C removal, each representing one metric ton of CO₂ that has been net removed from the atmosphere by the ecosystem during the monitoring period.

## Discussion

### The need for near real-time measurements

The EC method has been used for more than two decades mostly for research purposes around surface-atmosphere energy balance, focusing mainly on CO_2_ and H_2_O. This approach widely supports the understanding of ecosystem and climate processes and their roles in the C cycle [14]. Compared to biometric methods used for measuring CO_2_ exchange, based on spatially discrete measuring C in different pools throughout lover time, the EC method yields a continuous flux series that is unbiased, showing the maturity of the underlying science [30]. The steady outflow of data from EC systems and the advent of satellite internet have made near real-time reporting on C fluxes possible and integrable into C monitoring systems and emerging applications around climate change mitigation policies. The continuous tracking of net ecosystem CO₂ fluxes at high spatial resolution reinforces key calculations—such as those used in C accounting—and provides greater leverage for ecosystem monitoring efforts. Even though promising agreements have been reported on the calibration of EC measurements using remote sensing data. The approach is mainstream in C dynamics reconstructions despite the data lacks the near real-time component that is needed to properly quantify dynamics over time [47]. Moreover, remote sensing products at an adequate resolution (i.e., ~ 30 m) usually have low temporal resolution (i.e., 2–4 weeks) and can present problems with respect to topography (steep environments), vegetation cover (i.e., sparse vegetation challenge the signal to noise ratios implemented in i.e., the GEDI mission to estimate ground and canopy height elevation) and regarding the presence of persistence clouds that can create temporal gaps in optical imagery and also affect lidar measurements from space [48]. Therefore, having at least one flux tower installed in the ecosystem domain is key not only to have near real-time NEE estimates, but also to calibrate remote sensing products to be integrated into the analyses.

### Extrapolation of net ecosystem exchange measurements

After EC measurements are registered, the main challenge is their extrapolation outside the EC system footprint. The solution we propose to estimate NEE is based on the relations between GPP, and RECO with respect to surface temperature and soil water content. Our models, showing good agreement for GPP and RECO (R^2^ ≥ 0.80) and NEE (R^2^ = 0.67), were built using long-term records of optical imagery and EC measurements for northern Patagonia’s temperate forests where the availability of satellite images can be compromised from the presence of persistent adverse weather. However, the performance of the RECO model (R^2^ = 0.54) reflects the inherent complexity of modeling ecosystem respiration, which is influenced by belowground biological processes and microclimatic variation that are not easily captured by remote sensing proxies. Future work should incorporate additional soil and environmental variables to improve the predictive accuracy of this component.

Given that 70% of the tower’s flux footprint is confined to a 17.4-hectare area (see Supplementary Figure S8), the representativeness of the eddy covariance signal supports its use as a training reference for spatial extrapolation within eco-functional clusters. Methods for extrapolation of EC and NEE measurements may become available in the future (see Sect. "A conservation-oriented old-growth forest monitoring framework with local co-benefits"), but to the best of our knowledge, this is among the first attempts to extrapolate near real-time NEE estimates using satellite-derived predictors. Previously, Zhuravlev et al [49] developed global NEE estimation models using EC data calibrated with remote sensing and meteorological data for a wide variety of forest and non-forest cover types. They used the kernel ridge regression (KRR) algorithm—similar in nature to support vector machine (SVM)—to estimate and predict NEE at 30 m resolution. The reported accuracies of their NEE predictions for evergreen broadleaf forests—a biome comparable to our temperate evergreen forest conditions in Patagonia—yielded an R^2^ = 0.42 and an RMSE of 1.28 g C m^−2^ day^−1^ for their daily NEE estimates, and an R^2^ = 0.70 and an RMSE of 0.80 g C m⁻^2^ day⁻^1^ for their monthly NEE estimates. Our 8-day NEE model reached a R^2^ of 0.67 and an RMSE of 1.04 g C m^−2^ day^−1^, which falls within that performance range. Additional technical details on predictors, input variables, and model configuration are provided in Supplementary Section S4.4.

### A conservation-oriented old-growth forest monitoring framework with local co-benefits

Carbon markets need traceable and verifiable methods and datasets to conform additionality i.e., C absorbed from a timed and well-documented human intervention. This requirement originated under the Kyoto Protocol and, while well-intentioned, has been difficult to apply in conservation contexts. Old-growth forests store large amounts of C—globally and in Patagonia [29]—and contain irrecoverable and vulnerable biomass C [50], are now recognized for their ability to keep absorbing CO_2_ every year for centuries [51]. Moreover, this type of ecosystem provides other ecosystem services such as being habitat for biodiversity and are key to the water cycle. Their continued C uptake potential and ecological importance underscore the need for monitoring frameworks that move beyond traditional additionality requirements, enabling the protection of high-integrity ecosystems that would otherwise remain excluded from climate mitigation strategies.

The conservation-oriented monitoring model presented here supports the long-term protection of privately owned native forests with high ecological value through legally binding conservation easements. In parallel, it establishes the foundation for spatially explicit, data-driven C flux accounting supported by continuous measurements. This framework facilitates not only C balance assessments, but also future integration of biodiversity and hydrological monitoring indicators.

The C flux registry not only supports the scientific calculation of C sequestration but also enhances credibility, verification, and transparency in accounting C units. However, there are still some areas that are open for improvement in our model. One of them is to improve near real-time extrapolation of fluxes measured with flux towers. Due to the high cloudiness in the study area, it is unlikely that this can be done by using remote sensing products. More likely is to do this by integrating on-site measurements of flux-related variables, such as images from phenocams and micrometeorological variables that may be integrated into a mechanistic model. Additionally, complex topography may distort the satellite signal or bias model inputs, further limiting the reliability of spatial extrapolation in rugged terrain.

Compared to traditional C accounting approaches—primarily based on periodic forest inventories and static biomass maps—our integrative methodology offers several advantages. Conventional methods, while operationally simpler, often rely on generalized emission factors or limited temporal resolution, reducing their sensitivity to short-term changes or interannual variability. In contrast, our framework incorporates near real-time CO₂ flux data, structural baselines from airborne LiDAR, and dynamic remote sensing proxies, enabling continuous tracking and spatiotemporal extrapolation of C fluxes. This increases both the transparency and accuracy of C estimates. However, challenges remain regarding high data processing requirements and equipment costs, and the integration of heterogeneous datasets. Despite these limitations, we argue that in high-integrity ecosystems where precision and verification are paramount, the benefits of such an approach outweigh its complexities.

Scaling this framework to broader regions will require addressing technical challenges, such as processing high volumes of data and monitoring other relevant greenhouse gases associated with peatlands (methane) and agricultural ecosystems (nitrous oxide). Future research will also explore the integration of biodiversity monitoring in areas of global conservation importance, such as the Central Chile biodiversity hotspot.

Finally, to facilitate replication of the methodology that we propose, we emphasize that it relies on open-access datasets and standardized processing chains. For example, the canopy height model (CHM) from the META-WRI global dataset, used as an input to estimate aboveground biomass density, is publicly available. This allows other researchers to reproduce the analysis in different regions by applying similar procedures to those we used in Patagonia.

## Conclusions

This study presents a robust and replicable methodology to monitor forest C dynamics using near real-time CO₂ flux measurements and remote sensing technologies. The implementation of this model in sites under legally binding conservation instruments enhances its long-term applicability and relevance for conservation-oriented C monitoring.

The successful application of this methodology in Chilean Patagonia highlights the potential of old-growth forests as continuous C sinks and underscores the relevance of integrating ecosystem monitoring with policy-relevant mechanisms such as conservation easements and nature-based climate solutions. This near real-time and traceable C data can support transparent C accounting, inform land-use and conservation decisions, and strengthen climate policy frameworks at both national and international levels.

As this framework scales to broader geographies, future developments will include multi-gas integration (e.g., methane, N₂O), biodiversity monitoring, and improvements in the extrapolation of fluxes under data-limited conditions. These advancements will further contribute to global efforts in managing C stocks and informing mitigation strategies. Altogether, these innovations can support more inclusive and verifiable C monitoring systems that align scientific evidence with conservation and climate action goals.

## Supplementary Information

Additional file1

## Data Availability

The data supporting the findings of this study are confidential and cannot be publicly shared due to privacy agreements and landowner protections. However, processed datasets, scripts, and analytical codes used in the study can be made available upon reasonable request.
