# Supplementary material for: An integrative methodology to estimate high-resolution carbon stock and fluxes: a case study in the old-growth forests of the Chilean Patagonia
Source: Carbon Balance Manag. 2025 Dec 24;21:11. doi: 10.1186/s13021-025-00381-6 (PMC12794367; doi:10.1186/s13021-025-00381-6)
Supplement: Supplementary file 1 — Additional file1 [file 13021_2025_381_MOESM1_ESM.docx]

# **Supplementary Material**

**List of Acronyms**

The acronyms used throughout this supplementary document are listed below:

EC – Eddy Covariance

NEE – Net Ecosystem Exchange

NEP – Net Ecosystem Production

GPP – Gross Primary Productivity

RECO – Ecosystem Respiration

SIF – Solar-Induced Chlorophyll Fluorescence

RTSIF – Reconstructed TROPOMI SIF

NDVI – Normalized Difference Vegetation Index

NIR – Near-Infrared Reflectance

NIRv – Near-Infrared Reflectance of Vegetation Index

EFC – Eco-functional cluster

LST – Land Surface Temperature

LSWI – Land Surface Water Index

BT – Brightness Temperature

TOA – Top of the Atmosphere

TIR1 – Thermal Infrared 1 (Band)

Q2 – Second Quartile (Median)

CMDSGF – Configurable Marginal Distribution Sampling Gap Filling

Pv – Vegetation Proportion

LiDAR – Light Detection and Ranging

## **S1. Vegetation zones grouped by functional characteristics**

Table S1. Vegetation zones are grouped by functional characteristics.

| **Code** | **Vegetation belts** | **Vegetation zones grouped** |
| --- | --- | --- |
| P53 | Mediterranean deciduous forest of *Nothofagus obliqua - Persea lingue* | Mediterranean deciduous forest |
| P47 | Mediterranean interior deciduous forest of *Nothofagus obliqua - Cryptocarya alba* | Mediterranean interior deciduous forest |
| P60 | Mediterranean-Temperate Andean deciduous forest of *Nothofagus pumilio - N. obliqua* | Mediterranean-Temperate Andean deciduous forest |
| P58 | Temperate Andean deciduous forest of *Nothofagus alpina* - *Dasyphyllum diacanthoides* | Temperate Andean deciduous forest |
| P59 | Temperate Andean deciduous forest of *Nothofagus alpina* - *N. dombeyi* |  |
| P61 | Temperate Andean deciduous forest of *Nothofagus pumilio - Araucaria araucana* |  |
| P64 | Temperate Andean deciduous forest of *Nothofagus pumilio / Azara alpina* |  |
| P63 | Temperate Andean deciduous forest of *Nothofagus pumilio / Berberis ilicifolia* |  |
| P62 | Temperate Andean deciduous forest of *Nothofagus pumilio / Drimys andina* |  |
| P65 | Temperate Andean deciduous forest of *Nothofagus pumilio / Ribes cucullatum* |  |
| P56 | Temperate coastal deciduous forest of *Nothofagus alpina - Persea lingue* | Temperate coastal deciduous forest |
| P54 | Temperate deciduous forest of *Nothofagus obliqua - Laurelia sempervirens* | Temperate deciduous forest |
| P66 | Temperate-Antiboreal Andean deciduous forest of *Nothofagus pumilio / Maytenus disticha* | Temperate-Antiboreal Andean deciduous forest |
| P72 | Temperate coastal lauriphyllous forest of *Weinmannia trichosperma - Laureliopsis philippiana* | Temperate coastal lauriphyllous forest |
| P73 | Temperate interior lauriphyllous forest of *Nothofagus dombeyi - Eucryphia cordifolia* | Temperate interior lauriphyllous forest |
| P55 | Mediterranean-Temperate coastal mixed forest of *Nothofagus dombeyi - N. obliqua* | Mediterranean-Temperate coastal mixed forest |
| P88 | Temperate Andean mixed forest of *Nothofagus betuloides / Berberis ilicifolia* | Temperate Andean mixed forest |
| P89 | Temperate-Antiboreal Andean mixed forest of *Nothofagus betuloides - Nothofagus pumilio* | Temperate-Antiboreal Andean mixed forest |
| P76 | Mediterranean-Temperate Andean coniferous forest of *Araucaria araucana / Festuca scabriuscula* | Mediterranean-Temperate Andean coniferous forest |
| P75 | Temperate Andean coniferous forest of *Araucaria araucana - Nothofagus dombeyi* | Temperate Andean coniferous forest |
| P78 | Temperate Andean coniferous forest of *Fitzroya cupressoides* |  |
| P74 | Temperate coastal coniferous forest of *Araucaria araucana* | Temperate coastal coniferous forest |
| P77 | Temperate coastal coniferous forest of *Fitzroya cupressoides* |  |
| P79 | Temperate coastal coniferous forest of *Pilgerodendron uvifera - Tepualia stipularis* |  |
| P80 | Temperate coastal coniferous forest of *Pilgerodendron uvifera / Astelia pumila* |  |
| P90 | Antiboreal coastal evergreen forest of *Nothofagus betuloides - Embothrium coccineum* | Antiboreal coastal evergreen forest |
| P83 | Temperate Andean evergreen forest of *Austrocedrus chilensis - Nothofagus dombeyi* | Temperate Andean evergreen forest |
| P86 | Temperate Andean evergreen forest of *Nothofagus betuloides - Laureliopsis philippiana* |  |
| P87 | Temperate Andean evergreen forest of *Nothofagus betuloides / Chusquea macrostachya* |  |
| P82 | Temperate Andean evergreen forest of *Nothofagus dombeyi - Saxegothaea conspicua* |  |
| P81 | Temperate Andean evergreen forest of *Nothofagus dombeyi / Gaultheria phillyreifolia* |  |
| P85 | Temperate interior evergreen forest of *Nothofagus betuloides / Desfontainia fulgens* | Temperate interior evergreen forest |
| P84 | Temperate interior evergreen forest of *Nothofagus nitida - Podocarpus nubigenus* |  |
| P91 | Temperate-Antiboreal coastal evergreen forest of *Nothofagus betuloides - Drimys winteri* | Temperate-Antiboreal coastal evergreen forest |
| P123 | Mediterranean eastern steppe of *Festuca gracillima* | Mediterranean eastern steppe |
| P124 | Mediterranean eastern steppe of *Festuca gracillima / Mulinum spinosum* |  |
| P122 | Mediterranean-Temperate eastern steppe of *Festuca pallescens / Mulinum spinosum* | Mediterranean-Temperate eastern steppe |
| P125 | Temperate eastern steppe of *Festuca gracillima / Chiliotrichum diffusum* | Temperate eastern steppe |
| P121 | Antiboreal Andean forb vegetation of *Nassauvia pygmaea - N. lagascae* | Antiboreal Andean forb vegetation |
| P120 | Temperate Andean forb vegetation of *Nassauvia dentata - Senecio portalesianus* | Temperate Andean forb vegetation |
| P69 | Mediterranean-Temperate eastern deciduous shrubland of *Nothofagus antarctica / Berberis microphylla* | Mediterranean-Temperate eastern deciduous shrubland |
| P70 | Temperate-Antiboreal Andean deciduous shrubland of *Nothofagus antarctica / Chiliotrichum diffusum* | Temperate-Antiboreal Andean deciduous shrubland |
| P116 | Antiboreal Andean dwarf scrub of *Bolax gummifera - Azorella selago* | Antiboreal Andean dwarf scrub |
| P115 | Temperate Andean dwarf scrub of *Adesmia longipes - Senecio bipontinii* | Temperate Andean dwarf scrub |
| P114 | Temperate Andean dwarf scrub of *Discaria chacaye / Berberis empetrifolia* |  |
| P67 | Temperate Andean deciduous shrubland of *Nothofagus antarctica* | Temperate Andean deciduous shrubland |
| P68 | Temperate Andean deciduous shrubland of *Nothofagus antarctica / Empetrum rubrum* |  |
| P92 | Temperate coastal evergreen shrubland of *Pilgerodendron uvifera - Nothofagus nitida* | Temperate coastal evergreen shrubland |
| SV | No vegetation |  |
| P94 | Antiboreal coastal moorland of *Astelia pumila - Donatia fascicularis* | Antiboreal coastal moorland |
| P93 | Temperate coastal moorland of *Donatia fascicularis - Oreobolus obtusangulus* | Temperate coastal moorland |
| P95 | Temperate-Antiboreal coastal moorland of *Bolax caespitosus - Phyllachne uliginosa* | Temperate-Antiboreal coastal moorland |
| P96 | Temperate-Antiboreal interior moorland of *Sphagnum magellanicum / Schoenus antarcticus* | Temperate-Antiboreal interior moorland |

## **S2. Description of Sensors and Instruments Installed in the eddy covariance System.**

Table S2. Sensors and Instruments Installed in the eddy covariance System.

| **Instrument/Sensor** | **Brand/Model** | **Description** |
| --- | --- | --- |
| Infrared Gas Analyzer (“Enclosed”) | LI-COR / LI-7200RS | Concentration of Gases (CO_2_ & H_2_O) |
| 3D Ultrasonic Anemometer | Gill Instruments / WindMaster | 3D Wind Speed and Direction |
| Net Radiometer | Kipp & Zonen / NR Lite 2 | Net Radiation |
| 4-Component Net Radiometer | Hukseflux NR01 | Longwave Downward Radiation, Longwave Upward Radiation, Global Solar Radiation, Reflected Solar Radiation, and Net Radiation |
| Quantum Sensor | LI-COR / LI-190R | Photosynthetically Active Radiation (PAR) |
| Humidity and Temperature Probe | Vaisala / HMP155 | Air Temperature and Humidity |
| Tipping Bucket Rain Gauge | Texas Electronics / TR-525M | Precipitation |
| Pyranometer | LI-COR / LI-200R | Global Solar Radiation |
| Soil Heat Flux Plates | Hukseflux / HFP01SC | Soil Heat Flux |
| Soil Probes | Stevens / Hydra Probe II | Soil Moisture, Soil Temperature, and Electrical Conductivity |

## **S3. Calibrations, Configurations, Data Corrections and Post-Processing**

The calibration and configuration of sensors and instruments, along with necessary corrections, are vital to ensure data precision and reliability of the Eddy Covariance (EC) data collection. Each sensor is meticulously calibrated using manufacturer-provided parameters, which may require adjustment based on environmental and meteorological conditions over time.

Field setup considers installation factors such as proximity to obstacles, vertical placement, canopy height, wind direction, and exposure to environmental elements, with sensitivity parameters, formulas and units of measurements configured via Blueprint Utility software. Then, data is collected and synchronized via a data acquisition and retention modules (DAqM and DRM, LI-COR) and processed in near real-time using SmartFlux 2 and EddyPro software, which provides corrected flux and meteorological data, along with diagnostics. A minimum of two months of data collection is necessary for reliable carbon offset calculations, with monthly corrections following established methodologies to assess data consistency and quality.

Statistical analyses leverage techniques to identify and rectify anomalies or data errors, for example, in terms of basic operations for obtaining consistent data, configurations proposed by (Fratini & Mauder, 2014) and quality evaluation methods from (Foken & Wichura, 1996), focusing on stationarity and turbulence tests are used. Statistical assessments include (Mauder et al., 2013) algorithms and the nine statistical tests of (Vickers & Mahrt, 1997) for raw high-frequency data. These techniques address short-term outliers, amplitude resolution issues, drop-outs, absolute limits exceeded, statistical asymmetries, discontinuities, time lags, and thresholds exceeded, ensuring robust data processing. Wind data corrections include detecting non-stationary wind patterns, systematic variations, and ultrasonic anemometer inclination errors. Methods like the (Wilczak et al., 2001) double rotation (for carbon fluxes monitoring) and traditional planar fit methods (for carbon flux accounting) are employed to address these issues, ensuring accurate wind measurements, particularly in complex terrain. Spectral corrections address low- and high-frequency losses using high-pass and low-pass filters. Analytical corrections in the spectral domain, based on the methodologies of Moncrieff et al. (1997, 2004), are applied to compensate for flux attenuation caused by instrument configuration, sensor separation, time response limitations, finite averaging intervals, and detrending procedures, thereby improving the accuracy of flux estimates. Then, quality indicators are calculated for all fluxes based on a combination of partial indicators generated as a result of two widely adopted tests, which are described in detail in the literature (Foken et al., 2004; Foken & Wichura, 1996; Göckede et al., 2008). In EddyPro software, each of these tests provides a partial quality indicator ranging from "1" (high quality) to "9" (low quality), where then both indicators are combined using a quality norm designed by (Mauder & Foken, 2004), which is based on a 0-1-2 rating system (high, intermediate and low quality, respectively). Subsequently, during post-processing, the data is corrected in order to have only data with quality flags 0 or 1. In the case of footprint estimation, the (Kljun et al., 2004) methodology is used to identify the area contributing to flux measurements. The footprint itself is calculated in the field using the SmartFlux 2 system; however, the data exclusion process is carried out in the post-processing stage. Only data within a cumulative 70% contribution area are retained, excluding out-of-scope regions.

All this comprehensive process ensures high-quality, standardized data for scientific analysis. Next, post-processing evaluates and applies final corrections, reinforcing data integrity and enabling reliable evaluations.

### *S.3.1 Data post-processing*

Data post-processing is a critical step in refining the output from the SmartFlux 2 system and EddyPro software, enhancing data integrity and coherence by integrating multiple datasets. Sophisticated algorithms enable quality control and robust data analysis, facilitating accurate insights into ecosystem dynamics, CO_2_ fluxes, and climate interactions. Key actions include the application of a footprint analysis, which discards irrelevant fluxes, i.e., only data within the 70% cumulative contribution area were retained (Kljun et al., 2015) (see Supplementary Figure S8).

and Burba, (2013) recommendations for storage corrections that account for gas accumulation in complex canopies. The storage term of CO_2_ is calculated from the temporal changes in the gas concentration profile, more specifically by employing the integral from 0 to the measurement height of the partial derivative of CO_2_ concentration with respect to time (Montagnani et al., 2018). This storage value is then added to the flux obtained by the EC system to reach the final flux value. As a complementary quality method, the energy balance ratio (EBR) is calculated daily to assess measurement quality.

In the case of data gaps due to instrument issues, a bagging regression model (Breiman, 1996; Pedregosa et al., 2011) with a random forest as the base estimator was used to reconstruct missing values. This approach, similar to the methodology proposed by Zhu et al. (2022), is particularly suited for long gaps and accounts for complex, nonlinear relationships between fluxes and meteorological drivers (e.g., radiation, temperature, and VPD), ensuring consistency across the full time series.

Finally, net ecosystem exchange (NEE) is separated into gross primary productivity (GPP) and ecosystem respiration (RECO) using methodologies from (Reichstein et al., 2005) and (Lasslop et al., 2010). Once the post-processing stage is complete, the CO₂ flux data becomes the final dataset used for carbon flux accounting and assessment of carbon balance consistency across time and space, ensuring a comprehensive understanding of ecosystem processes in response to environmental changes.

## The final dataset includes one complete year of EC measurements (15 July 2024 – 15 July 2025). Out of the total, 92% of the data were successfully recorded. After quality filtering, 26.5% of the dataset was gap-filled using the bagging regression approach described above. The net ecosystem exchange (NEE) was adjusted using storage correction and EBR consistency and was partitioned into GPP and RECO. The annual NEE flux was −4.0 Mg CO₂ ha⁻¹ year⁻¹ with a propagated standard deviation (SD) of 0.206 Mg CO₂ ha⁻¹ year⁻¹, which represents the total uncertainty of the annual sum of fluxes.

## **S.4 Expanded Methods for CO₂ Flux Monitoring and Spatial Scaling (Section 2.5)**

### *S.4.1 Spatial Extrapolation of CO_2_ Fluxes*

While the EC methodology is currently the most scientifically validated technology for measuring CO_2_ fluxes, its measurement-footprint is constrained. It measuring range typically covers 200-1000 m from the measurement point, depending on the tower elevation and locally specific meteorological and topographical characteristics. Accordingly, statistical tools were used to extrapolate CO_2_ flux measurements to areas with similar functional characteristics (defined here as the Eco-functional clusters, (EFC). The EC system directly measures the NEE of CO_2_, which is assumed to be similar to net ecosystem production (NEP) and can therefore be estimated as the difference between two fluxes: GPP (Baldocchi, 2020) and RECO (Raich & Schlesinger, 1992) both derivable from satellite remote sensing.

### *S.4.2 Gross Primary Production (GPP) modeling*

We modelled GPP using recent advancements in remote sensing that establish a direct relationship between GPP and solar-induced chlorophyll fluorescence (SIF), an electromagnetic signal emitted by chlorophyll-a during solar energy absorption (Zhang & Peñuelas, 2023). SIF serves as an immediate indicator of photosynthetic activity, responding to environmental stressors and effectively tracking GPP across various forest ecosystems (Guanter et al., 2012; Porcar-Castell et al., 2014, Pascual et al., 2021). Linear regression models were generated to predict GPP in the study area every 8 days at a spatial resolution of 30 m, utilizing SIF and GPP satellite products with coarser resolutions (5 km) over the period 2001-2019 (Table 1). The SIF dataset, termed ‘RTSIF’, was created by Chen et al. (Chen et al., 2022b) using the Extreme Gradient Boosting (XGBoost) algorithm, which reconstructs TROPOMI SIF data by integrating satellite products such as Land Surface Temperature (LST) and photosynthetically active radiation. The final ‘RTSIF’ products were validated against the original TROPOMI SIF product and were compared to SIF measurements obtained from tower-installed sensors and the OCO-2 and GOME-2 SIF products. The GPP dataset (Table 1) by Zhang et al. (Zhang et al., 2017b) was developed from an improved light use efficiency model based on MODIS data and validated against GPP data from 113 EC towers in the FLUXNET network. The linear regressions between SIF and GPP were constructed as linear fits with an intercept at the origin and were separated by season to account for the variability in the phenology of evergreen forests:

$GPP = \alpha\times SIF$ ***Eq. 1***

where *GPP* is the gross primary productivity (g C m^-2^ day^-1^), *SIF* is the solar-induced chlorophyll fluorescence (mW m^-2^ µm^-1^ sr^-1^) and *α* is the 1^st^ parameter of the model (g C µm sr mW^-1^ day^-1^).

Once the GPP prediction models were constructed using SIF data, a downscaling method increased the spatial resolution of the ‘RTSIF’ products from 5 km to 30 m. The near-infrared reflectance of vegetation (NIRv) index derived from satellite images was used for this purpose (see Equation 2), as it is considered as (Badgley et al., 2017):

$NIR_{v} = NIR\times\left( NDVI-0.08 \right)$ ***Eq. 2***

where *NIRv is* the near-infrared reflectance of vegetation index, *NIR* is the near-infrared reflectance and *NDVI –* 0.08 is the modified normalized difference vegetation index, where 0.08 is subtracted to minimize the influence of bare soil.

The NIRv index was estimated using satellite images from NASA's Harmonized Landsat and Sentinel-2 (HLS) project, offering a spatial resolution of 30 m (Masek et al., 2021). Specifically, HLS L30 products from Landsat 8 and 9 were employed for the period 2013-2020, utilizing the B05 band for NIR and calculating NDVI as the normalized difference between B05 (NIR) and B04 (red). Spectral bands were averaged to produce 8-day interval products, aligning with the 5 km resolution GPP and SIF satellite products. Then, a modification of the downscaling algorithm proposed by Turner et al. (Turner et al., 2020) was used (see Equation 3), incorporating a delta factor (ΔSIF) to address deviations between the coarse scale ‘RTSIF’ product and the finer SIF product, allowing the smoothing of extreme values:

$SIF_{30m} = \left( SIF_{5km}\times\frac{NIR_{v}}{\overline{NIR_{v}}} \right)+\Delta SIF$ ***Eq. 3***

where *SIF_30m_* is the high-resolution (30 m) solar induced chlorophyll fluorescence (mW m^-2^ um^-1^ sr^-1^), *SIF_5km_* is a 5-km coarser estimate of the afore-said variable, *NIRv* is the near-infrared reflectance of vegetation index, $\overline{NIRv}$ is the mean near-infrared reflectance of vegetation index and Δ*SIF* is the adjustment factor for mean deviations (mW m^-2^ um^-1^ sr^-1^).

The ΔSIF factor (see Equation 4) was derived from the difference between the coarse scale ‘RTSIF’ product and a SIF raster of the same spatial resolution (5 km), obtained by mean-resampling a 30-m SIF (see Equation 5). This last 30 m SIF raster was generated directly with the Turner et al. (Turner et al., 2020) algorithm, which does not incorporate the ΔSIF factor.

$\Delta SIF=SIF_{5km}-SIF_{5km\left( 30m \right)}$ ***Eq. 4***

where *ΔSIF* is the adjustment factor for mean deviations (mW m^-2^ um^-1^ sr^-1^), *SIF_5km_* is the low-resolution (5 km) Solar Induced Chlorophyll Fluorescence (mW m^-2^ um^-1^ sr^-1^), *SIF_5km(30m)_* is the low-resolution (5 km) Solar Induced Chlorophyll Fluorescence, resampled from the mean values of high-resolution (30 m) SIF (mW m^-2^ um^-1^ sr^-1^).

$SIF_{5km\left( 30m \right)} = Resample_{mean}\left( SIF_{5km}\times\frac{NIR_{v}}{\overline{NIR_{v}}} \right)$ ***Eq. 5***

Where *SIF_5km(30m)_* is the low-resolution (5 km) Solar Induced Chlorophyll Fluorescence, resampled from the mean values of high-resolution (30 m) SIF (mW m^-2^ um^-1^ sr^-1^). Factors such as the ΔSIF factor are commonly applied in remote sensing for downscaling continuous variables (Atkinson, 2013; Kustas et al., 2003). conversion factors were then applied to convert flux units from GPP units (g C m⁻² day⁻¹) to ton CO_2_ ha⁻¹ year⁻¹. Finally, the satellite-based GPP estimates were validated using a linear regression constrained to the origin. The validation was conducted against ground-based GPP measurements from the CL-SDF site. A total of 64 data pairs were used, each representing an 8-day average. For each pair, the satellite-derived GPP was computed as the mean of all pixels located within the historical footprint of the EC system. Prior to analysis, outliers were removed using a Hampel filter.

### *S.4.3 Ecosystem respiration (RECO) modeling*

For modeling RECO, various remote sensing models use driver variables such as air temperature, LST, vegetation spectral indices, and GPP, relations that have been validated in different scientific studies such as (Jägermeyr et al., 2014) and (Wu et al., 2014). Notably, the model selected for this study was developed by Bazzi et al. (2024), which closely resembles the ReRSM model (Gao et al., 2015) (applied to mountain shrublands and mixed temperate forests) and incorporates soil moisture, linked to microbial respiration in soils (Cook & Orchard, 2008). The algorithm was modified to replace soil moisture with the Land Surface Water Index (LSWI):

$RECO=\left( R_{0}+k\times GPP \right)\times\left( \frac{LSWI}{LSWI_{max}+LSWI} \right)\times e^{E_{0}\left( \frac{1}{T_{ref}-T_{0}}-\frac{1}{LST-T_{0}} \right)}$ ***Eq. 6***

where *RECO* is the ecosystem respiration (g C m^-2^ day^-1^), *R_0_* is the parameter of ecosystem reference respiration at reference temperature (g C m^-2^ day^-1^), *k* is the parameter associated with the autotrophic respiration fraction of the ecosystem, *GPP* is the gross primary productivity (g C m^-2^ day^-1^), *LSWI* is the land surface water index, *LSWI_max_* is the maximum land surface water index for the period, *E_0_* is the parameter of activation energy, *T_ref_* is the reference soil temperature (283.15 °K, equivalent to 10 °C), *T_0_* is the minimum soil temperature for respiration to occur (227.13 °K, equivalent to -46.02 °C) and *LST* is the land surface temperature (°K).

To obtain indexes LSWI, LSWI_max_ and LST, spectral bands from HLS L30 images were averaged to produce 8-day interval products, aligning temporally with the 30-m GPP rasters (see section 2.4.1). The formula for LSWI is provided in Equation 9, with LSWI_max_ calculated as the maximum LSWI value between 2013 and 2020.

$LSWI=\frac{\left( NIR-SWIR2 \right)}{\left( NIR+SWIR2 \right)}$ ***Eq. 7***

where *LSWI* is the land surface water index, *NIR* is the near-infrared reflectance and *SWIR2* is the shortwave infrared reflectance 2.

The method proposed by Avdan & Jovanovska, (2016) was used to estimate LST (Eq. 8)

$LST=\frac{BT_{TIR1}}{\left\{ 1+\left[ \left( \frac{\lambda BT}{\rho} \right)\times ln\varepsilon_{\lambda} \right] \right\}}$ ***Eq. 8***

where *LST* is the land surface temperature (°K), *BT_TIR1_* is the brightness temperature at the top of the atmosphere, derived from the thermal infrared 1 (*TIR1*) band (K), *λBT* is the wavelength of emitted brightness temperature, derived from the thermal infrared 1 (TIR1) band (10.895 x 10^-6^ m), ρ is the relation factor between Boltzmann constant, Planck constant and the speed of light (1.438 x 10^-2^ m K), and *Ɛ_λ_* is the Surface emissivity.

The brightness temperature (BT) at the top of the atmosphere (TOA) was obtained from the B10 spectral band of the HLS L30 product, calculated in °C and then converted to °K. Surface emissivity was determined from the NDVI value, following the criteria established by (Avdan & Jovanovska, 2016) (see Equation 9).

***Eq. 9***


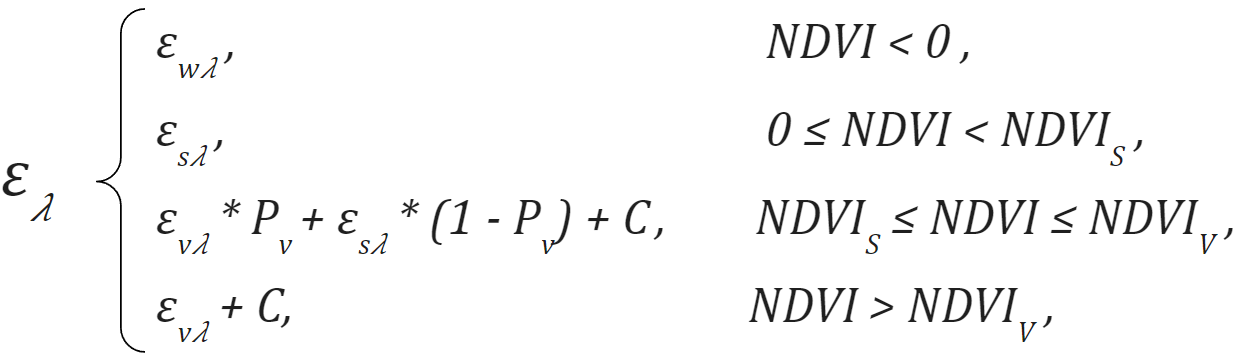


Where *Ɛ_λ_* is the Soil surface emissivity, *Ɛ_wλ_* is the Water emissivity (0.991), *Ɛ_sλ_* is the bare soil emissivity (0.966), *Ɛ_vλ_* is the vegetation emissivity (0.973), *P_v_* is the vegetation proportion, *C* is the soil roughness (0.005), *NDVI* is the normalized difference vegetation index, *NDVI_S_* is the NDVI for bare soil (0.2) and *NDVI_V_* is the NDVI for vegetation (0.5).

The NDVI index was calculated from the HLS L30 product by normalizing the difference between the B05 (NIR) and B04 (red) bands. The vegetation proportion (P_v_) was then determined using Equation 10.

$P_{v}=\left( \frac{NDVI-NDVI_{s}}{NDVI_{v}-NDVI_{s}} \right)^{2}$ ***Eq. 10***

where *NDVI* is the normalized difference vegetation index, *NDVI_S_* is the NDVI for bare soil (0.2), and *NDVI_V_* is the NDVI for vegetation only (0.5).

After calculating the necessary variables for the RECO model, parameters were estimated using 8-day LSWI data (2013-2020), EC-based estimates of GPP and field-measured soil temperature (2014-2022) obtained at the CL-SDF AmeriFlux site. Estimates for GPP and soil temperature data were averaged in 8-day intervals to match the LSWI temporal resolution. Initial parameters values were based on literature regarding EC measurements in similar ecosystems (Perez‐Quezada et al. 2018; 2023) and were subsequently optimized using a nonlinear least-squares regression. Once the optimized parameters were obtained, the RECO model (Equation 8) was spatialized with satellite data, generating RECO products every 8 days at 30-m resolution. Conversion factors were then applied to convert flux units from g C m⁻² day⁻¹ to ton CO_2_ ha⁻¹ year⁻¹. Finally, a validation of the satellite model was conducted by a linear regression with an intercept at the origin between the observations and predictions for RECO using 64 coinciding data pairs, where each satellite register corresponds to the mean RECO of pixels within the historical footprint of the EC system.

### *S.4.4 Net Ecosystem Exchange of CO_2_ (NEE) estimation and validation*

Our NEE products were generated every 8 days at a 30 m resolution using Equation 13 and the GPP and RECO rasters for the period 2013-2020 as inputs. Negative NEE values indicate CO_2_ capture or sequestration, while positive values represent CO_2_ emissions. Model performance was evaluated using a linear regression constrained to the origin, comparing modeled NEE values with ground-based NEE measurements from the EC AmeriFlux BASE CL-SDF site in northern Patagonia. A total of 56 matching 8-day data pairs were used. For each pair, the satellite-derived NEE was calculated as the mean of all pixels falling within the historical footprint of the eddy covariance system. Outliers were excluded prior to analysis using a Hampel filter, consistent with the approach applied to GPP.

$NEE=RECO-GPP$ ***Eq. 11***

where *NEE* is the net ecosystem exchange (µmol CO_2_ m^-2^ s^-1^), *RECO* is the ecosystem respiration (µmol CO_2_ m^-2^ s^-1^), and *GPP* is the gross primary productivity (µmol CO_2_ m^-2^ s^-1^).

As a methodological reference, Zhuravlev et al. (2022) developed global models for estimating net ecosystem exchange (NEE) using eddy covariance (EC) data calibrated with satellite and meteorological variables. Their models employed the kernel ridge regression (KRR) algorithm—a non-linear method comparable to support vector machines (SVMs)—to estimate NEE at 30 m spatial resolution. Predictor variables included Landsat-derived spectral indices (from Landsat 5, 7, and 8), band ratios, and scaled meteorological variables from the AgERA5 dataset, including air and dew-point temperature, precipitation, and surface solar radiation.

Their model evaluation for evergreen broadleaf forests, the biome most analogous to our temperate evergreen forests in Patagonia, yielded R² = 0.42 and RMSE = 1.28 g C m⁻² day⁻¹ for daily estimates, and R² = 0.70 and RMSE = 0.80 g C m⁻² day⁻¹ for monthly estimates.

In our case, we employed a simpler linear model constrained to the origin, calibrated for 8-day average fluxes using GPP and RECO maps derived from remote sensing, and validated against EC measurements at the CL-SDF site. Our NEE model yielded R² = 0.67 and RMSE = 1.04 g C m⁻² day⁻¹, which compares favorably to the range reported by Zhuravlev et al. (2022). Although our model uses a more transparent and replicable approach, its performance supports the validity of using satellite-based predictors to upscale NEE in complex ecosystems such as Chilean temperate forests.

### *S.4.5 Weighting factor for spatial extrapolation of CO_2_ fluxes.*

At least one EC tower in each EFC to measure CO_2_ fluxes, along with a meteorological station to monitor microclimatic, energy balance and soil variables. Using EC data, the footprint or representative polygon for CO_2_ flux measurements is calculated using the Kljun method (Kljun et al., 2004), enabling the determination of a weighting factor for spatially extrapolating field-measured data to other land properties within the EFC. This is, from the modeled NEE data representing the historical mean (2013-2020) (section 2.5.3), the footprint pixels are extracted and compared with the pixels of all land properties within the EFC as a spatial mask. This is done by estimating the ratio between the second quartile (Q2, or median) of both datasets (see Equation 12). The median is chosen to make the formula applicable to datasets with different types of distribution. The weighting factor (*P_Ext_*) is then applied to the carbon flux estimates as a common coefficient for all land properties within the project area located inside the EFC.

$P_{Ext}=\frac{Q2\left( NEE_{EFC} \right)}{Q2\left( NEE_{f} \right)}$ ***Eq. 12***

where *P_Ext_* is the spatial extrapolation weighting factor, *Q2(NEE_EFC_)* is the 2nd quartile or median of NEE (2013-2020) data for land properties within the EFC, and Q*2*(*NEE_f_*) is the 2^nd^ quartile or median of NEE (2013-2020) data for the EC tower footprint.

Since new land properties can be incorporated into our platform at any time, this calculation is performed every six months to obtain a calibrated weight factor based on the extension of current land properties.

Data processing for all modeling, spatial, and statistical analyses was performed using R and R Studio (R Core Team, 2024), using the *terra* (Hijmans et al., 2024) and *ggplot2* (Wickham, 2016) libraries for visualizations. Maps were generated using the QGIS 3.34.1 software.

## **S5. LiDAR 3D and Tree ID.**


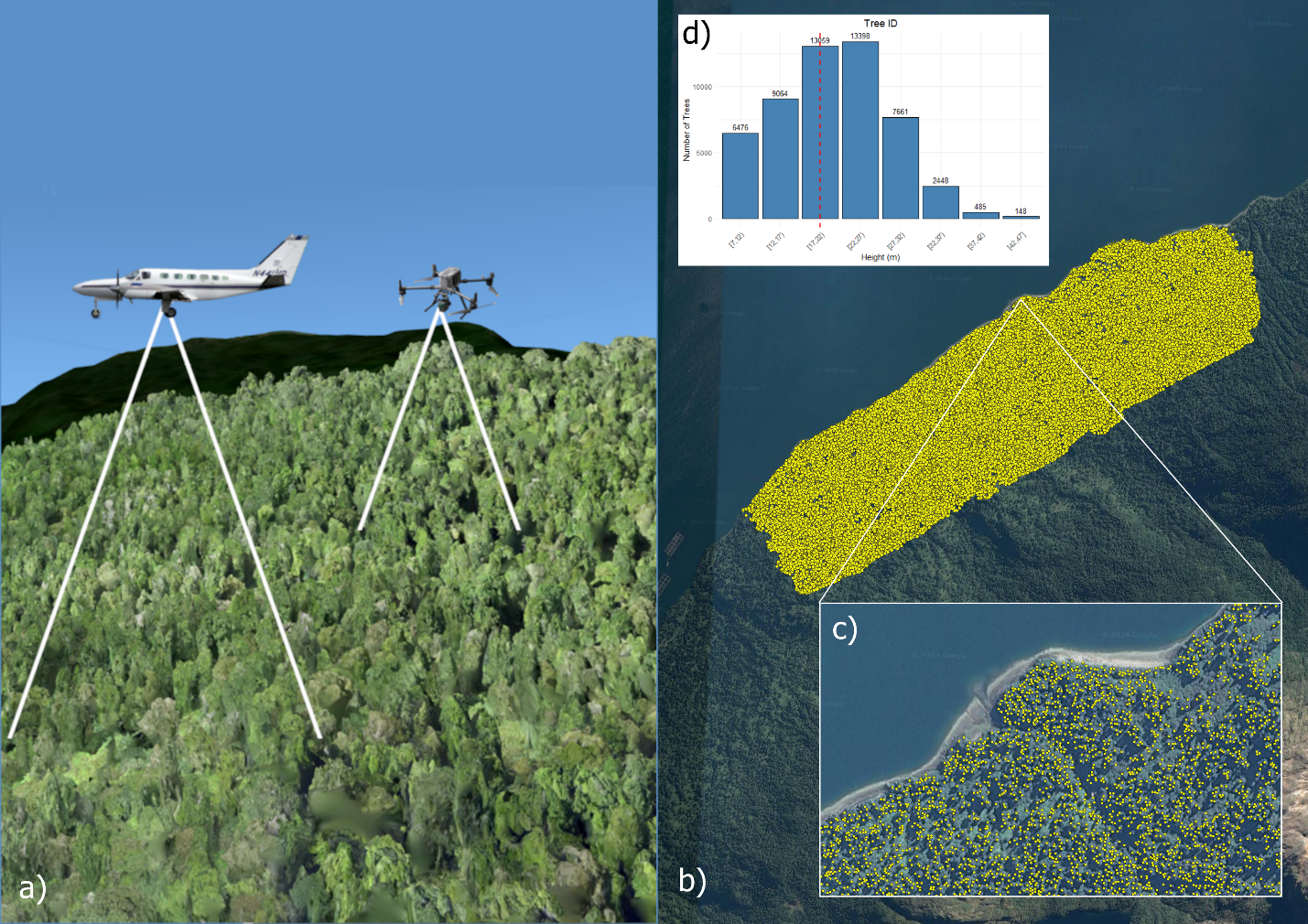


**Figure S5.** Example of the 3D model generated using LIDAR technology, along with the tree count conducted in the baseline using Tree ID. a) Representation of different types of LiDAR surveys: one conducted from an airplane and the other by a drone. b) yellow points represent all the trees identified by the algorithm for individual tree detection. c) Zoomed-in view of the detected trees. d) Graph showing the distribution of tree heights versus the number of trees. In the selected area, there are 52,739 trees, with an average height ranging between 17 and 22 meters.

## **S6. Height-to-biomass regression model used to estimate aboveground biomass density (AGBD) from canopy height.**


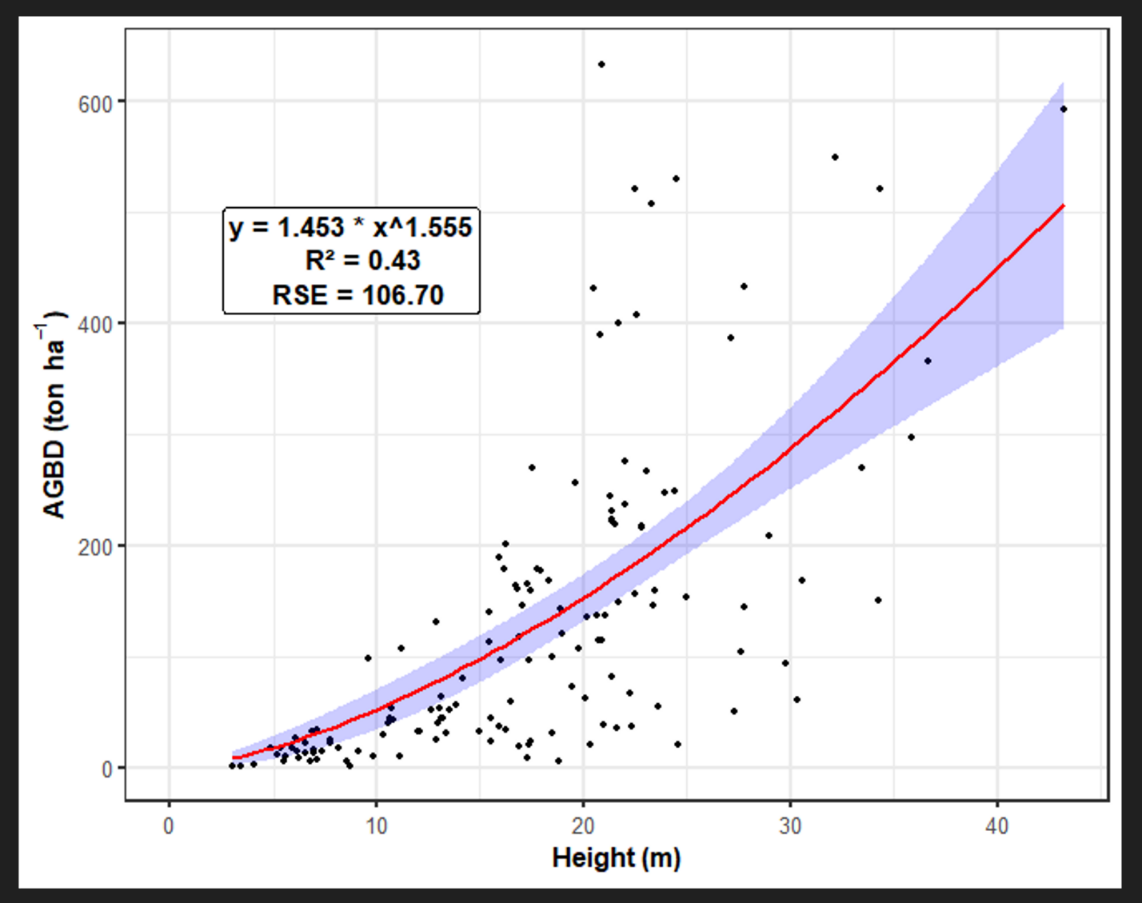


**Figure S6.** Relationship between canopy height (m) and aboveground biomass density (AGBD, Mg ha⁻¹) based on field plot data used to calibrate the height-to-biomass model. The red line represents the fitted power-law regression model (AGBD = 1.453 × Height^1.555), with the shaded area indicating the 95% confidence interval. The coefficient of determination (R² = 0.43) and residual standard error (RSE = 106.70) reflect the model’s performance.

## **S7. Distributions of canopy height and aboveground biomass density from UAV and META-WRI data (pixels ≥ 4 m in height).**

Figure S7 -a illustrates the frequency distribution of tree height, while Figure S7-b displays the frequency distribution of AGBD (Mg ha⁻¹). All AGBD data were calculated only for pixels with a height ≥ 4 m, as this was the filter used to estimate biomass equivalent to forest.


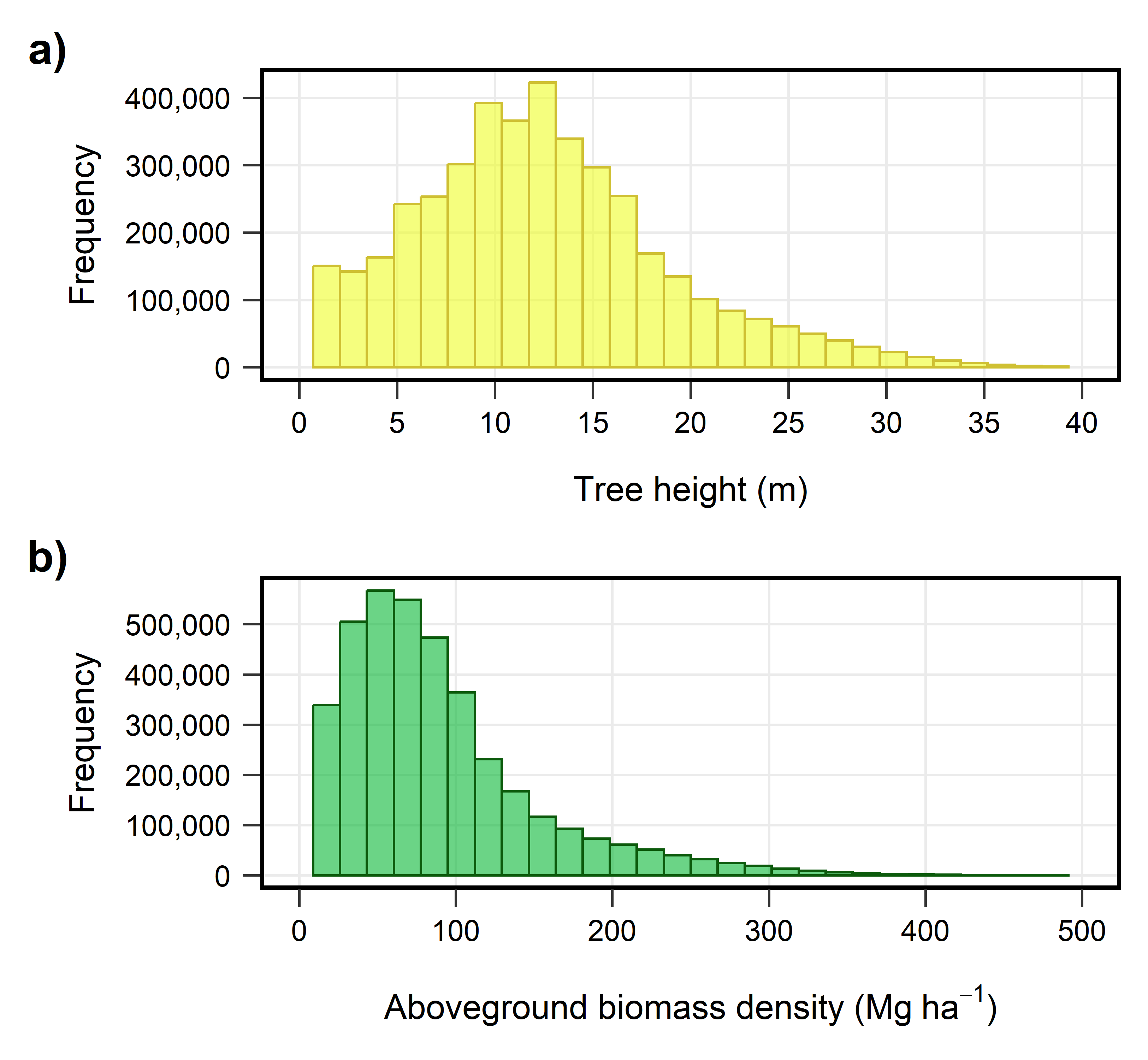


**Figure S7.** (a) Canopy Height Model frequency and (b) Aboveground biomass density frequency in the Conservation Easement land areas in the platform.

## **S8. Eddy Covariance system area of influence.**


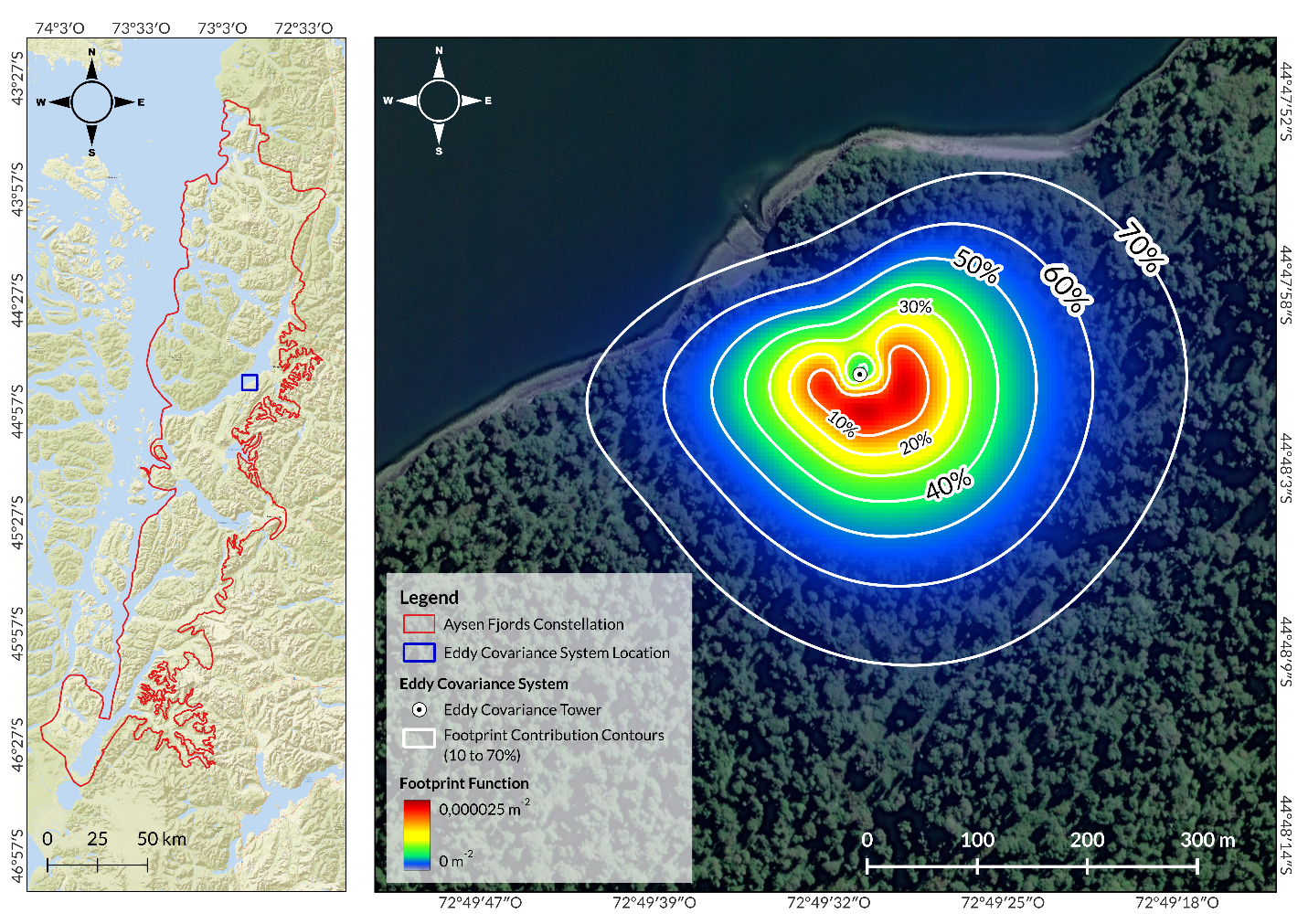


**Figure S8.** Showcase of an Eddy Covariance system located in the Aysén Fjords over a temperate rainforest, showing the area of influence of 17.4 ha.

**References**

Atkinson, P. M. (2013). Downscaling in remote sensing. International Journal of Applied Earth Observation and Geoinformation, 22, 106–114. https://doi.org/https://doi.org/10.1016/j.jag.2012.04.01

Avdan, U., & Jovanovska, G. (2016). Algorithm for automated mapping of land surface temperature using LANDSAT 8 satellite data. Journal of Sensors, 2016(1), 1480307. https://doi.org/https://doi.org/10.1155/2016/1480307

Badgley, G., Field, C. B., & Berry, J. A. (2017). Canopy near-infrared reflectance and terrestrial photosynthesis. Science Advances, 3(3), e1602244. https://doi.org/DOI:10.1126/sciadv.16022

Baldocchi, D. D. (2020). How eddy covariance flux measurements have contributed to our understanding of Global Change Biology. Global Change Biology, 26(1), 242–260. https://doi.org/https://doi.org/10.1111/gcb.14807

Bazzi, H., Ciais, P., Abbessi, E., Makowski, D., Santaren, D., Ceschia, E., Brut, A., Tallec, T., Buchmann, N., & Maier, R. (2024). Assimilating Sentinel-2 data in a modified vegetation photosynthesis and respiration model (VPRM) to improve the simulation of croplands CO2 fluxes in Europe. International Journal of Applied Earth Observation and Geoinformation, 127, 103666. <https://doi.org/https://doi.org/10.1016/j.jag.2024.103666>

Breiman, L. 1996. Bagging predictors, Machine Learning, 24, 123–140.

Burba, G. (2013). Eddy covariance method for scientific, industrial, agricultural and regulatory applications: A field book on measuring ecosystem gas exchange and areal emission rates. LI-Cor Biosciences.

Chen, X., Huang, Y., Nie, C., Zhang, S., Wang, G., Chen, S., & Chen, Z. (2022b). A long-term reconstructed TROPOMI solar-induced fluorescence dataset using machine learning algorithms. Scientific Data, 9(1), 427. https://doi.org/https://doi.org/10.1038/s41597-022-01520-1

Cook, F. J., & Orchard, V. A. (2008). Relationships between soil respiration and soil moisture. Soil Biology and Biochemistry, 40(5), 1013–1018. https://doi.org/https://doi.org/10.1016/j.soilbio.2007.12.012

Foken, T., & Wichura, B. (1996). Tools for quality assessment of surface-based flux measurements. Agricultural and Forest Meteorology, 78(1–2), 83–105. https://doi.org/https://doi.org/10.1016/0168-1923(95)02248-1

Foken, T., Göockede, M., Mauder, M., Mahrt, L., Amiro, B., & Munger, W. (2004). Post-field data quality control. In Handbook of micrometeorology: a guide for surface flux measurement and analysis (pp. 181–208). Springer. https://doi.org/https://doi.org/10.1007/1-4020-2265-4_9

Fratini, G., & Mauder, M. (2014). Towards a consistent eddy-covariance processing: an intercomparison of EddyPro and TK3. Atmospheric Measurement Techniques, 7(7), 2273–2281. https://doi.org/https://doi.org/10.5194/amt-7-2273-2014

Gao, Y., Yu, G., Li, S., Yan, H., Zhu, X., Wang, Q., Shi, P., Zhao, L., Li, Y., & Zhang, F. (2015). A remote sensing model to estimate ecosystem respiration in Northern China and the Tibetan Plateau. Ecological Modelling, 304, 34–43. https://doi.org/https://doi.org/10.1016/j.ecolmodel.2015.03.001

Göckede, M., Foken, T., Aubinet, M., Aurela, M., Banza, J., Bernhofer, C., Bonnefond, J.-M., Brunet, Y., Carrara, A., & Clement, R. (2008). Quality control of CarboEurope flux data–Part 1: Coupling footprint analyses with flux data quality assessment to evaluate sites in forest ecosystems. Biogeosciences, 5(2), 433–450. https://doi.org/https://doi.org/10.5194/bg-5-433-2008

Guanter, L., Frankenberg, C., Dudhia, A., Lewis, P. E., Gómez-Dans, J., Kuze, A., Suto, H., & Grainger, R. G. (2012). Retrieval and global assessment of terrestrial chlorophyll fluorescence from GOSAT space measurements. Remote Sensing of Environment, 121, 236–251. https://doi.org/https://doi.org/10.1016/j.rse.2012.02.006

Hijmans, R. J., Bivand, R., Forner, K., Ooms, J., Pebesma, E., & Sumner, M. D. (2024). terra: Spatial Data Analysis; 2022. URL Https://CRAN. R-Project. Org/Package= Terra. R Package Version, 1–6.

Jägermeyr, J., Gerten, D., Lucht, W., Hostert, P., Migliavacca, M., & Nemani, R. (2014). A high‐resolution approach to estimating ecosystem respiration at continental scales using operational satellite data. Global Change Biology, 20(4), 1191–1210. https://doi.org/https://doi.org/10.1111/gcb.12443

Kljun, N., Calanca, P., Rotach, M. W., & Schmid, H. P. (2004). A simple parameterisation for flux footprint predictions. Boundary-Layer Meteorology, 112, 503–523. https://doi.org/https://doi.org/10.1023/B:BOUN.0000030653.71031.96

Kljun, N., Calanca, P., Rotach, M. W., and Schmid, H. P. (2015). A simple two-dimensional parameterization for Flux Footprint Prediction (FFP), Geoscientific Model Development, 3695-3713, https://doi.org/10.5194/gmd-8-3695-2015

Kustas, W. P., Norman, J. M., Anderson, M. C., & French, A. N. (2003). Estimating subpixel surface temperatures and energy fluxes from the vegetation index–radiometric temperature relationship. Remote Sensing of Environment, 85(4), 429–440. https://doi.org/https://doi.org/10.1016/S0034-4257(03)00036-1

Lasslop, G., Reichstein, M., Papale, D., Richardson, A. D., Arneth, A., Barr, A., Stoy, P., & Wohlfahrt, G. (2010). Separation of net ecosystem exchange into assimilation and respiration using a light response curve approach: critical issues and global evaluation. Global Change Biology, 16(1), 187–208. https://doi.org/https://doi.org/10.1111/j.1365-2486.2009.02041.x

Luebert, F., & Pliscoff, P. (2017). Pisos vegetacionales de Luebert y Pliscoff 2017.

Mauder, M., & Foken, T. (2004). Documentation and instruction manual of the eddy-covariance software package324 TK3. Abt. Mikrometeorol., 46.

Mauder, M., Cuntz, M., Drüe, C., Graf, A., Rebmann, C., Schmid, H. P., Schmidt, M., & Steinbrecher, R. (2013). A strategy for quality and uncertainty assessment of long-term eddy-covariance measurements. Agricultural and Forest Meteorology, 169, 122–135. https://doi.org/https://doi.org/10.1016/j.agrformet.2012.09.006

Masek, J., Ju, J., Roger, J., Skakun, S., Vermote, E., Claverie, M., Dungan, J., Yin, Z., Freitag, B., Justice, C. (2021). HLS Operational Land Imager Surface Reflectance and TOA Brightness Daily Global 30m v2.0 [Data set]. NASA EOSDIS Land Processes Distributed Active Archive Center. Accessed 2025-05-02 from https://doi.org/10.5067/HLS/HLSL30.002

Moncrieff, J. B., Massheder, J. M., De Bruin, H., Elbers, J., Friborg, T., Heusinkveld, B., Kabat, P., Scott, S., Soegaard, H., & Verhoef, A. (1997). A system to measure surface fluxes of momentum, sensible heat, water vapour and carbon dioxide. Journal of Hydrology, 188, 589–611. https://doi.org/https://doi.org/10.1016/S0022-1694(96)03194-0

Moncrieff, J., Clement, R., Finnigan, J., & Meyers, T. (2004). Averaging, detrending, and filtering of eddy covariance time series. In Handbook of micrometeorology: A guide for surface flux measurement and analysis (pp. 7–31). Springer. https://doi.org/https://doi.org/10.1007/1-4020-2265-4_2

Montagnani, L., Grünwald, T., Kowalski, A., Mammarella, I., Merbold, L., Metzger, S., Sedlák, P., & Siebicke, L. (2018). Estimating the storage term in eddy covariance measurements: the ICOS methodology. International Agrophysics, 32(4), 551–567. https://doi.org/DOI:10.1515/intag-2017-0037

Pascual, A., Giardina, C. P., Selmants, P. C., Laramee, L. J., & Asner, G. P. (2021). A new remote sensing-based carbon sequestration potential index (CSPI): A tool to support land carbon management. Forest Ecology and Management, 494, 119343.

Perez‐Quezada, J. F., Celis‐Diez, J. L., Brito, C. E., Gaxiola, A., Nuñez‐Avila, M., Pugnaire, F. I., & Armesto, J. J. (2018). Carbon fluxes from a temperate rainforest site in southern South America reveal a very sensitive sink. Ecosphere, 9(4), e02193. https://doi.org/https://doi.org/10.1002/ecs2.2193

Perez‐Quezada, J. F., Moncada, M., Barrales, P., Urrutia‐Jalabert, R., Pfeiffer, M., Herrera, A. F., & Sagardía, R. (2023). How much carbon is stored in the terrestrial ecosystems of the Chilean Patagonia? Austral Ecology, 48(5), 893–903. https://doi.org/https://doi.org/10.1111/aec.13331

Porcar-Castell, A., Tyystjärvi, E., Atherton, J., Van der Tol, C., Flexas, J., Pfündel, E. E., Moreno, J., Frankenberg, C., & Berry, J. A. (2014). Linking chlorophyll a fluorescence to photosynthesis for remote sensing applications: mechanisms and challenges. Journal of Experimental Botany, 65(15), 4065–4095. https://doi.org/https://doi.org/10.1093/jxb/eru191

R Core Team (2024). R: A language and environment for statistical computing. R Foundation for Statistical Computing, Vienna, Austria. URL https://www.R-project.org/.

Raich, J. W., & Schlesinger, W. H. (1992). The global carbon dioxide flux in soil respiration and its relationship to vegetation and climate. Tellus B, 44(2), 81–99. https://doi.org/https://doi.org/10.1034/j.1600-0889.1992.t01-1-00001.x

Reichstein, M., Falge, E., Baldocchi, D., Papale, D., Aubinet, M., Berbigier, P., Bernhofer, C., Buchmann, N., Gilmanov, T., & Granier, A. (2005). On the separation of net ecosystem exchange into assimilation and ecosystem respiration: review and improved algorithm. Global Change Biology, 11(9), 1424–1439. https://doi.org/https://doi.org/10.1111/j.1365-2486.2005.001002.x

Turner, A. J., Köhler, P., Magney, T. S., Frankenberg, C., Fung, I., & Cohen, R. C. (2020). A double peak in the seasonality of California’s photosynthesis as observed from space. Biogeosciences, 17(2), 405–422. https://doi.org/https://doi.org/10.5194/bg-17-405-2020

Vickers, D., & Mahrt, L. (1997). Quality control and flux sampling problems for tower and aircraft data. Journal of Atmospheric and Oceanic Technology, 14(3), 512–526. https://doi.org/https://doi.org/10.1175/1520-0426(1997)014<0512:QCAFSP>2.0.CO;2

Wickham, H. (2016). ggplot2: elegant graphics for data analysis Springer-Verlag New York; 2009. Preprint At, 2, 15545–15550.

Wilczak, J. M., Oncley, S. P., & Stage, S. A. (2001). Sonic anemometer tilt correction algorithms. Boundary-Layer Meteorology, 99, 127–150. https://doi.org/https://doi.org/10.1023/A:1018966204465

Wu, C., Gaumont-Guay, D., Black, T. A., Jassal, R. S., Xu, S., Chen, J. M., & Gonsamo, A. (2014). Soil respiration mapped by exclusively use of MODIS data for forest landscapes of Saskatchewan, Canada. ISPRS Journal of Photogrammetry and Remote Sensing, 94, 80–90. https://doi.org/https://doi.org/10.1016/j.isprsjprs.2014.04.018

Zhang, Y., Xiao, X., Wu, X., Zhou, S., Zhang, G., Qin, Y., & Dong, J. (2017). A global moderate resolution dataset of gross primary production of vegetation for 2000–2016. Scientific Data, 4(1), 1–13. https://doi.org/https://doi.org/10.1038/sdata.2017.165

Zhang, Y., & Peñuelas, J. (2023). Combining solar-induced chlorophyll fluorescence and optical vegetation indices to better understand plant phenological responses to global change. Journal of Remote Sensing, 3, 0085. https://doi.org/DOI:10.34133/remotesensing.0085

Zhu, S., Clement, R., McCalmont, J., Davies, C. A., and Hill, T. (2022). Stable gap-filling for longer eddy covariance data gaps: A globally validated machine-learning approach for carbon dioxide, water, and energy fluxes, *Agr. Forest Meteorol.,* 314, 108777 https://doi.org/10.1016/j.agrformet.2021.108777.
